# Do dogs preferentially encode the identity of the target object or the location of others’ actions?

**DOI:** 10.1007/s10071-024-01870-w

**Published:** 2024-03-30

**Authors:** Lucrezia Lonardo, Christoph J. Völter, Robert Hepach, Claus Lamm, Ludwig Huber

**Affiliations:** 1 Comparative Cognition, Messerli Research Institute, University of Veterinary Medicine of Vienna, Medical University of Vienna and University of Vienna, Veterinärplatz 1, Vienna, 1210 Austria; 2 Department of Comparative Cultural Psychology, Max Planck Institute for Evolutionary Anthropology, Leipzig, 04103 Germany; 3 Department of Experimental Psychology, University of Oxford, Oxford, OX2 6GG UK; 4 Social, Cognitive and Affective Neuroscience Unit, Department of Cognition, Emotion and Methods in Psychology, Faculty of Psychology, University of Vienna, Vienna, 1010 Austria

**Keywords:** Social cognition, Dog cognition, Action perception, Goal-directed actions, Eye-tracking

## Abstract

The ability to make sense of and predict others’ actions is foundational for many socio-cognitive abilities. Dogs (*Canis familiaris*) constitute interesting comparative models for the study of action perception due to their marked sensitivity to human actions. We tested companion dogs (*N* = 21) in two screen-based eye-tracking experiments, adopting a task previously used with human infants and apes, to assess which aspects of an agent’s action dogs consider relevant to the agent’s underlying intentions. An agent was shown repeatedly acting upon the same one of two objects, positioned in the same location. We then presented the objects in swapped locations and the agent approached the objects centrally (Experiment 1) or the old object in the new location or the new object in the old location (Experiment 2). Dogs’ anticipatory fixations and looking times did not reflect an expectation that agents should have continued approaching the same object nor the same location as witnessed during the brief familiarization phase; this contrasts with some findings with infants and apes, but aligns with findings in younger infants before they have sufficient motor experience with the observed action. However, dogs’ pupil dilation and latency to make an anticipatory fixation suggested that, if anything, dogs expected the agents to keep approaching the same location rather than the same object, and their looking times showed sensitivity to the animacy of the agents. We conclude that dogs, lacking motor experience with the observed actions of grasping or kicking performed by a human or inanimate agent, might interpret such actions as directed toward a specific location rather than a specific object. Future research will need to further probe the suitability of anticipatory looking as measure of dogs’ socio-cognitive abilities given differences between the visual systems of dogs and primates.

**Supplementary Information:**

The online version contains supplementary material available at 10.1007/s10071-024-01870-w.

Interdisciplinary investigators have addressed the question of how we understand what others are doing when we watch them perform goal-directed actions. A limited system for representing agents’ actions as purposeful is probably present already in infancy (Spelke 2022). While the developmental trajectory of action understanding is well documented (e.g., Hunnius and Bekkering 2014), studying dogs (*Canis familiaris*) can shed light on the cognitive requirements underlying this ability. Indeed, dogs do not perform typical human actions themselves, but still successfully respond to our actions (such as the pointing gesture; Kaminski and Nitzschner 2013).

Two main paradigms have been employed to study the development of infants’ expectations about others’ goal-directed actions. In violation of expectation paradigms (Woodward 1998), infants are habituated to an event showing an agent grasping one of two objects, positioned in the same location across habituation trials (e.g., always the object on the right side of the scene). Subsequently, the sides of the objects are swapped. Finally, in test trials, the agent reaches for the “old identity/new side” object (i.e., the object that they had reached for during habituation, now on the new side of the scene) or the “old side/new identity” object (the object that was ignored during habituation, now on the side of the scene that was reached for during habituation). The dependent variable was the infants’ cumulative looking time at the test events. A longer looking time is thought to reveal violations of expectations that infants develop during habituation (e.g., Margoni et al. 2022; Stahl and Kibbe 2022; Woodward 1998; but see Paulus 2022).

In the anticipatory looking paradigm (Cannon and Woodward 2012), infants are familiarized with a fixed number of trials showing similar events to those described above. The crucial difference is that, in anticipatory looking designs, the test events (after the locations of the objects have been swapped) are paused after the agent has started to centrally approach the two objects. Therefore, participants’ anticipatory looks are used as a measure of what they predict the agent will reach for, based on what participants observed during the familiarization.

Two studies found that infants’ looking times and anticipatory gaze shifts reflect an expectation that a human hand and arm (but not a mechanical claw) will continue approaching the old identity object in the new location (Cannon and Woodward 2012; Woodward 1998). These results have also been replicated and extended to actions in which infants cannot perform themselves, as long as an (inanimate) agent shows certain features in its behaviour, such as equifinal variations towards the target, efficient movements and a salient action effect (e.g., Biro and Leslie 2007; Csibra 2008; Luo 2011; Luo and Baillargeon 2005; Southgate et al. 2008).

However, other studies have found null results or support for the expectation that an animated agent will continue approaching the same location even if it now hosts a different object relative to the familiarization/habituation phase (Daum et al. 2012; Ganglmayer et al. 2019). The contrast between the results by Daum et al. (2012) and Cannon and Woodward (2012) does not seem to depend on the agent (an animated fish vs. a recorded human arm) performing the action (Ganglmayer et al. 2019). While there is evidence that great apes in the anticipatory looking paradigm predict that a human hand will continue grasping the old identity object and that a mechanical claw will grasp the (new) object on the old side (Kano and Call 2014), the original findings (Cannon and Woodward 2012) could not be replicated in a study with infants Ganglmayer et al. (2019).

Learning based on observational experience with everyday actions (such as hands grasping objects) might facilitate action prediction even for actions that the observer cannot actively perform. Hunnius and Bekkering (2014) suggested that, in making sense of others’ actions, younger infants – with limited motor abilities - might rely on their observational experience. As their motor system develops, infants might rely increasingly on first-person motor experience with the observed actions. It has therefore been suggested that action understanding might depend on the interplay between observational and motor experiences and that researchers should try to assess how these processes interact (Woodward and Gerson 2014).

During the course of their ontogeny in human households, companion dogs gain extensive observational experience with human actions, but lack the ability to perform those actions directly. This is a crucial difference from the great apes studied thus far (e.g., Kano and Call 2014; Myowa-Yamakoshi et al. 2012), who might have had both observational and motor experience with the actions on which they were tested. In contrast, dogs likely lack the experience in carrying out the human actions shown in our experiments (grasping with a hand and kicking); however unlike human infants, they can be tested once their motor system is fully developed and hence can help clarify the specificity (Southgate 2013) of the first-person motor experience needed for identifying others’ action goals. Previous research with infants (Kanakogi and Itakura 2011; Krogh-Jespersen and Woodward 2018; Sommerville et al. 2005) suggested that, in this task, first-person motor experience is relevant for interpreting the human agent’s behaviour during familiarization as being guided by the “intention to reach for the previously approached object”. In the present study, we tested whether a species that lacks motor experience with the observed actions still shows infant-like expectations that human agents continue approaching the old identity object even along a new path.

There is already evidence that dogs pay attention to human goal-directed actions and are able to replicate them through imitation or emulation (Fugazza et al. 2019; Fugazza and Miklósi 2014; Huber et al. 2009; Topál et al. 2006). Interestingly, when confronted with a choice between matching the identity of the object manipulated by a demonstrator or the spatial location where the demonstration took place, dogs were found to preferentially match the location rather than the target (Fugazza et al. 2016). These results partially conflict with the findings of a previous study in which dogs were tested on Woodward’s paradigm, using real-life stimuli (Marshall-Pescini et al. 2014). In this setting, dogs looked longer when a human agent changed the object she interacted with rather than the location in the room she had approached relative to the habituation phase. When the agent was instead an inanimate box, the dogs did not show differential looking times to the “new identity” and “new side” events.

In addition to the role of observational and motor experience in action understanding, another point remains unsettled from studies employing Woodward’s paradigm. Namely, it is unclear whether infants consistently show a novelty response to the agents’ behaviour (operationalized as longer dwell times to the old side/new identity event) or active prediction of the agent’s action (operationalized as predictive gaze shifts from the agents to the old identity object). For this reason, in our second experiment, we collected data on both parameters (the results of the dwell times are in the SM).

However, as a cumulative measure collected after the test events have already unfolded (Aslin 2007), dwell times do not allow us to evaluate specifically which features of the stimuli elicited surprise or interest (Sirois and Jackson 2012). Pupillometry (Hepach, forthcoming; Mathôt 2018; Sirois and Brisson 2014 for overviews) has been proposed as a more sensitive (Jackson and Sirois 2009) and complementary measure to cumulative looking times for the quantification of surprise in Violation of Expectation (VoE) paradigms, provided that luminosity differences are controlled for. Indeed, measuring pupil size allows researchers to assess the time course of participants’ attention during the unfolding of the events (Jackson and Sirois 2022; Sirois and Jackson 2012). Moreover, as a continuously varying response, pupil size allows us to make inferences about graded psychological constructs. For example, changes in pupil size were found to co-vary with the magnitude of prediction errors (Nassar et al. 2012). However, when measuring both dwell times and pupil size, it remains necessary to clearly specify the hypothesis linking the data to their explanatory cognitive construct (Aslin 2012).

For these reasons, during test events, in addition to the dogs’ dwell time, we measured their phasic pupil size changes as a complementary measure of prediction error (i.e., the detection of a discrepancy between expectations and perceptual input), as previously described in other studies with dogs (Völter et al. 2023; Völter and Huber 2021, 2022) and humans (Zhang and Emberson 2020). This is the first study measuring pupil size in a Woodward-style goal attribution paradigm in dogs; the use of pupillometry in this task has recently been established with infants in a multi-lab collaboration (Sirois et al. 2023).

In the first experiment of the present study, we used a paradigm previously used with human infants (Cannon and Woodward 2012; Ganglmayer et al. 2019) and great apes (Kano and Call 2014) to assess whether dogs generate anticipatory looks to an agent’s target object (i.e., the object upon which the agent is about to act). In the second experiment, we additionally showed the final phase of the agent’s approach during the test trial. Therefore we applied a violation of expectation paradigm, but unlike in previous studies (Marshall-Pescini et al. 2014; Woodward 1998, 1999), we did not habituate dogs to the events, to optimise our task for the measurement of anticipatory looks and changes in pupil size. In one condition, the agent approached the new object on the old side (old side/new identity condition), while in the other the agent approached the old object on the new side (old identity/new side condition). The agents were a human and an inanimate but self-propelled box, approaching and kicking one of two toys. We chose this action because it minimized the differences between the movements of the human and inanimate agents and because there is already evidence that dogs can predict the target a human is about to kick (Lonardo et al. 2023). Specifically, the current study builds on our previous findings indicating that dogs pay attention to approaching, grasping and kicking actions, and that they look at the target object faster if the action is performed by a human than by a conspecific. The inanimate agent in Experiment 2 lacked a face and biological motion but appeared self-propelled (similarly to the inanimate agent in Marshall-Pescini et al. 2014).

Based on previous studies with dogs (Marshall-Pescini et al. 2014), infants (Cannon and Woodward 2012; Woodward 1998) and apes (Kano and Call 2014), we had hypothesized that dogs preferentially encode the identity of the object when attending to actions. Therefore we expected longer dwell times to and larger pupil sizes for the old side/new identity event than for the old identity/new side event. Additionally, if dogs form the expectation that the agents continue approaching the same object as during the familiarization, before the agent starts moving in the test trial we expected them to generate predictive gaze shifts toward the object that the agent had acted upon during the familiarization.

Furthermore, if the presence of certain features of the agent or the action (such as self-propulsion, choice between two possible objects, or the salient action effect; see Elsner and Adam 2021) are sufficient to generate the impression of agency, we expected dogs’ looking patterns to be similar between the human and the inanimate agent conditions. If, instead, action prediction also depends on the saliency of the agent (Lonardo et al. 2023), we would expect differences between the two agents. Specifically, we had predicted that the inanimate agent might have captured dogs’ attention more than the human agent, given dogs’ unfamiliarity with self-propelled inanimate objects.

## Experiment 1 – methods

### Subjects

Thirteen dogs (5 females) of different breeds were tested in this experiment. The mean age at the beginning of the experiment was 36 ± 15 months. Table S1 reports demographic and counterbalancing details.

**Fig. 1:**
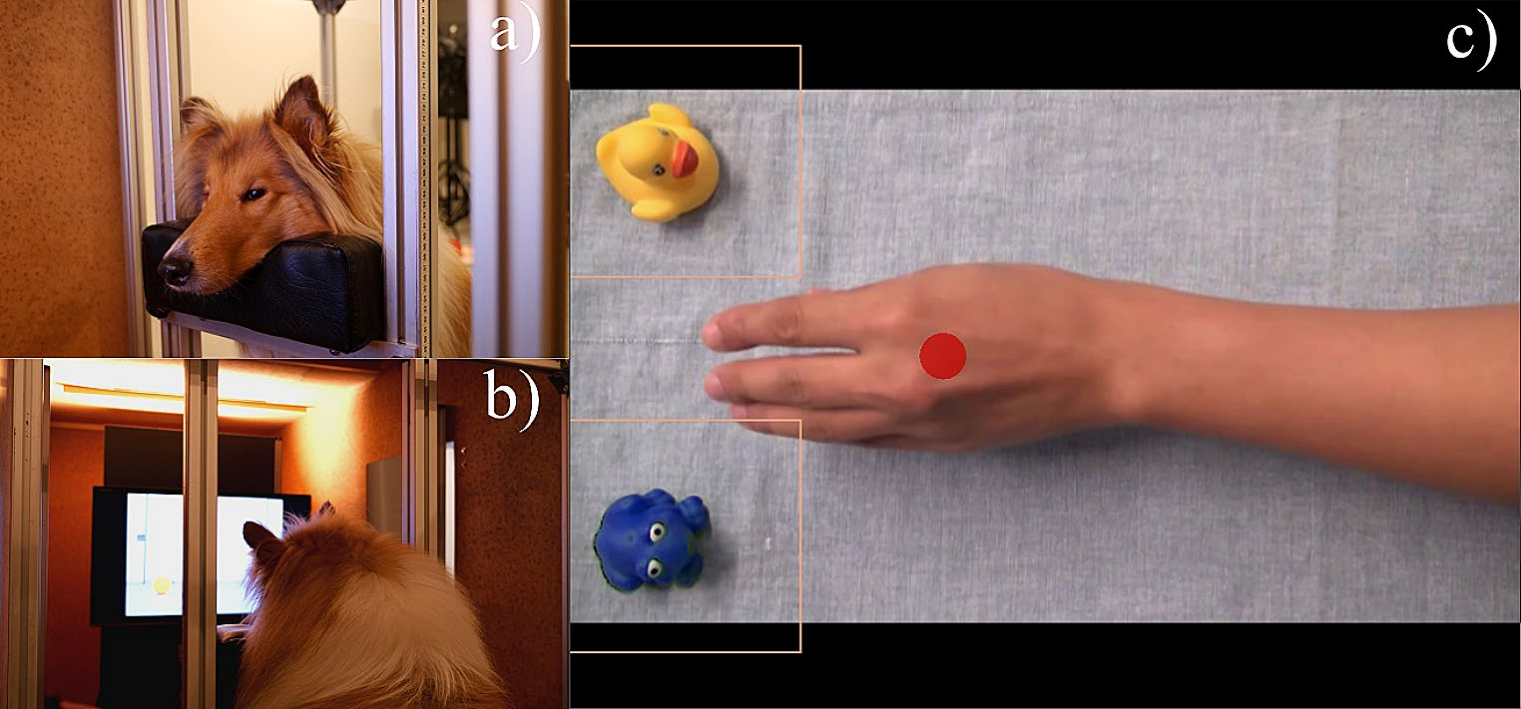
Set- up and stimuli for Experiment 1. A dog lays its head on the chinrest while looking at the screen (**a-b**). A screenshot from one of the test sessions (**c**). Pink squares indicate the objects AoIs; the red dot shows a dog’s estimated central focus of attention

For both experiments, the sample size was determined by the number of chinrest- and calibration-trained dogs available at the time of data collection (but we discuss the power in the SM and in the Results section of Experiment 2). The criterion for successful training was that dogs perform a validation of a calibration with less than 1° of visual angle deviation and are able to maintain their head on the chinrest (without leaving when the trainer left their field of view) for up to one minute (Fig. 1a-b). On average, dogs needed approximately 13 (± 5) training sessions.

### Stimuli

We used the same 15-s-long videos as Kano and Call (2014), but we displayed them without sounds (during piloting we found that sounds drew dogs’ attention off the screen). Moreover, we digitally edited the colour of the frog (Fig. 1c), to ensure that dogs could clearly perceive the difference between the two objects (a blue frog and a yellow duck). Videos were displayed at a frame rate of 25 Hz on an LCD monitor (resolution: 1024 × 768 px), contingent on the dogs’ gaze being detected for 200 ms in the centre of the screen. The video area occupied 1024 × 576 px on the screen (32° x 18° of visual angle). In each video a hand or a mechanical claw was recorded from above approaching from the right side of the screen always the same one of two objects. The objects were positioned one in the upper and one in the lower left part of the screen. After the agent had grasped the target object three times (familiarization), the positions of the objects were swapped, the agent was shown approaching the objects centrally and the video ended when the agent was immobile and equidistant from the two objects (test). Between events, a grey screen was presented for 250 ms. We considered as areas of interest (AoIs) squares, centred around the two objects, with a side of 250 px (Fig. 1c).

### Design and procedure

**Fig. 2:**
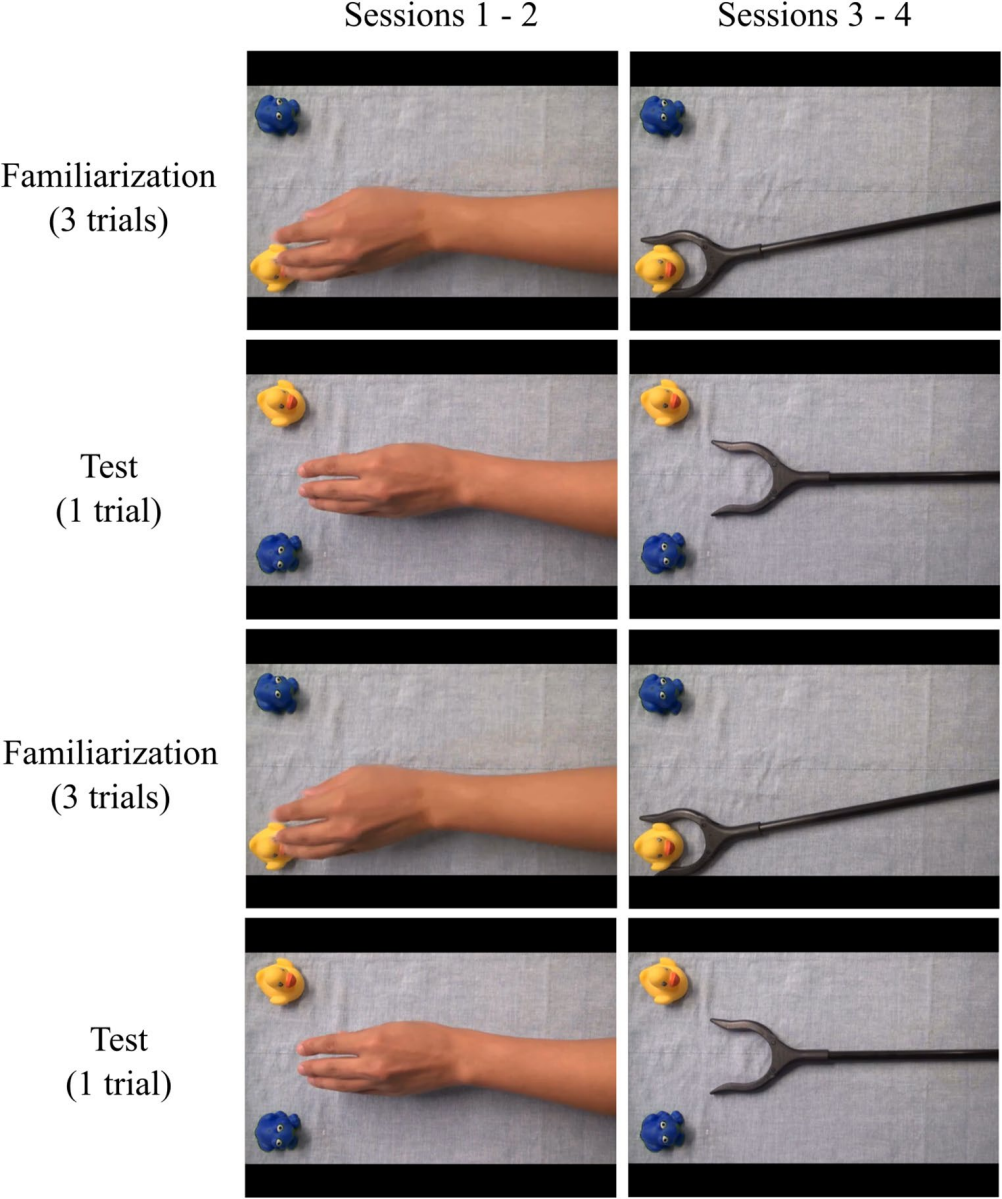
Experimental design of Experiment 1 (we used the video stimuli by Kano and Call 2014, with modified coloring of one of the target objects)

We replicated as closely as possible Kano & Call’s (2014) experimental design, with the exception of a lower number of sessions, due to limitations in our dog caregivers’ availability. Specifically, in a within-subject design, each dog was tested in four sessions. Each session consisted of two identical repetitions of the sequence: three familiarization trials followed by one test trial. Hence, in total, dogs were presented with four test trials for each of the two agents (hand and claw); the order of presentation of the agents was blocked and which agent was presented in the first two sessions was counterbalanced across the sample. The identity of the target object (duck or frog) was counterbalanced between subjects while their position during familiarization trials was kept constant: the duck in the lower part of the screen and the frog in the upper part (and vice versa during test trials; Fig. 2).

On testing days, dogs laid their head on the chinrest (height adjusted depending on the dog’s size) and their right eye was tracked at 1000 Hz using an EyeLink 1000 eye-tracker (SR Research, Canada). The dog’s eyes were approx. 70 cm away from the screen. Each session began with a five-point calibration of each dog’s gaze positions on the screen. We used animations (size: 64 px) to attract the dogs’ attention to each calibration point.

### Data parsing

To reparse eye-movement events, we used the “Slow/offline” algorithm of Data Viewer (*EyeLink Data Viewer*, 2021), which, by taking into account more samples in the calculation, generates more reliable values than the EyeLink online parser when estimating the velocity and acceleration of eye-movements post-hoc. For the pupillometry and the familiarization analyses, we instead worked with the raw data.

### Statistical analyses

All the statistical analyses were conducted in R (R Core Team 2022; version 4.2.2) and *p*-values less than 0.05 were used as inference criteria to establish statistical significance. For all the models, if convergence issues were encountered, we removed the correlation parameter(s) between the random intercept and slope(s) and if needed we simplified the random slope structure (for more details in the Supplementary Materials; SM 1).

### Proportion of dwell time with respect to the old identity object

The interest period (IP) for this analysis consisted of the approximately 3 s from the moment the hand or claw reached the centre between the two objects until the end of the video. We calculated the proportion of dwell time to the old-identity object (i.e., the object that was grasped during the familiarization) over the total dwell time to both objects. Trials in which the dogs did not look at any of the objects during the IP were assigned a proportion of dwell time equal to zero for this analysis. To analyse the effect of the agent on the proportion of time spent looking at the old-identity object, we fitted a generalized linear mixed model (GLMM; Baayen 2008) with beta error structure and logit link function (Bolker 2008; McCullagh and Nelder 1989), using the R-function *glmmTMB* of the homonymous package (Brooks et al. 2017; version 1.1.5). We transformed the distribution of the dependent variable to exclude the exact values of 0 and 1 because the beta distribution is not defined for these extreme values (Smithson and Verkuilen 2006). We included in the model the agent (human or inanimate) as only test predictor, and the agent shown first, the session number (1 to 4, z-transformed) and the location of the target object (upper or lower part of the screen) as control predictors. We additionally included the random slope of session number and agent (centred) within subject ID. The correlation parameters between random slopes and intercept were not included.

Additionally, we fitted an intercept-only model to test whether the proportion of dwell time to the old-identity object significantly differed from the chance level (0.5). Trials in which the dogs did not look at any of the objects during the IP were excluded from this analysis. As control predictor with fixed effects, we added the z-transformed trial number as well as the random slope of the trial number within the subject ID. A parameter for the correlation between the random slope and intercept was not included.

### First fixation

During the same IP described in the section above, we scored whether the dogs’ first fixation was directed to the old-identity object (scored as 1) or to the old-side object (scored as 0). Trials in which the dogs did not fixate on any of the two AoIs within the IP were also scored as zeros. For this dependent variable, we fitted a GLMM with a binomial error structure and logit link function (McCullagh and Nelder 1989). We used the same fixed and random effects as in the model above and included the correlations between random slopes and intercepts.

## Experiment 1 – results

### Proportion of dwell time with respect to the old identity object

The average proportion of dwell time to the old identity object was 0.43 (SD ± 0.49) and to the old side object 0.57 (SD ± 0.49), when the agent was human. When the agent was inanimate, the average proportion of dwell time to the old identity object was 0.56 (SD ± 0.49) and to the old side object 0.44 (SD ± 0.49). There was no significant difference between the hand and claw trials with respect to the proportion of dwell times to the old-identity object (Table S5). Dogs showed a preference to look at the old-identity object when this was shown in the upper part of the screen (χ^2^_1_ = 7.71, *p =* .005), irrespective of the agent shown.

The proportion of dwell time to the old identity object was not significantly different from 0.5 (Wald test: z = -0.16, *p* = .874) and was not affected by the trial number (Table S6).

### First fixation

Aggregating the data across dogs and trials, on average 27 (SD ± 45)% of the dogs fixated on the old identity object first when the agent was human and 29 (SD ± 46)% when the agent was inanimate; 29 (SD ± 46) % of the dogs fixated on the old side object first when the agent was human and 21 (SD ± 41) % when the agent was inanimate. The remaining dogs did not make anticipatory fixations. The probability that dogs fixated the old identity object first was not affected by the agent. Rather, dogs were more likely to direct their first fixation to the old identity object when it was in the upper part of the screen (χ^2^_1_ = 8.55, *p* = .003; Table S7). Dogs also tended to fixate first the old side object when this was in the upper part of the screen (likelihood ratio test: χ^2^_1_ = 3.73; *p* = .053) but their probability of fixating the old side object first did not depend on the agent (likelihood ratio test: χ^2^_1_ = 0.63; *p* = .426). The probability of directing the first fixation to the old identity object first did not differ significantly from chance (Table S8).

## Experiment 1 – discussion

The present eye-tracking experiment tested dogs on an adaptation of the paradigms by Cannon and Woodward (2012) and Kano and Call (2014). We asked whether, after a three-trial exposure to a reaching action, dogs, like infants and apes, would come to perceive the identity of the grasped object as a human agent’s goal and the location where the action took place as the inanimate agent’s goal. To this end, we used stimuli comparable to those used in previous developmental (Cannon and Woodward 2012; Ganglmayer et al. 2019) and comparative (Kano and Call 2014) studies. Unlike great apes and human 11-month-old infants, dogs lack the ability to execute the human movements shown in these videos; hence, dogs can help clarify whether anticipatory fixations in this task crucially depend on motor experience with the observed movements, as suggested by previous research (Krogh-Jespersen and Woodward 2018). If motor experience with the observed actions is necessary to predict actions in this task, dogs should not be able to generate anticipatory looks in the way human infants and great apes did.

When the agents stopped centrally between the two objects, dogs did not predict with their first fixation that a human hand should have continued approaching the old identity object in the new location. Furthermore, unlike infants and great apes, dogs’ first fixations did not reflect an expectation that the inanimate claw should reach for the old location, which contains the new object. Rather, dogs fixated more often whichever object was shown in the upper part of the screen, possibly because of the chinrest. Consistent with their first fixations, the dogs’ proportion of dwell time did not differ between the AoIs of the two objects and was not influenced by the animacy of the agent.

These results resemble those of 8-month-old infants who did not receive an active training with reaching actions (Krogh-Jespersen and Woodward 2018) and are in accordance with the hypothesis that the ability to interpret others’ actions as goal-directed in this task requires a corresponding motor ability. However, it is also possible that dogs failed to (encode and) visually predict others’ action goals due to the excessive speed of the stimuli, which were sped up compared to the ones in Cannon and Woodward (2012). During familiarization trials (see SM), dogs did not use the agents’ trajectory to predict with their gaze the target object (i.e., the object the agent was about to grasp). Instead, their gaze arrival times to the target object were neither significantly predictive nor reactive, indicating that the dogs were following the movement of the agents. When the agent was inanimate, their gaze arrival times to the distractor (i.e., the object that was not approached by the claw) were significantly predictive. This suggests that when the agent is inanimate dogs visually explore the scene more often or look less at the agent.

The scarcity of anticipatory looks observed in this experiment might be due to the fast hand movements shown in the videos and to dogs’ decreased familiarity with viewing only human hands from a bird’s eye perspective. The possibility of seeing the agents’ whole bodies, might play an important role in dogs’ perception of humans (Boch et al. 2023; Correia-Caeiro et al. 2021). Furthermore, it is possible that dogs did not recognize the movement shown in the video as occurring on a horizontal surface, since the monitor was vertical, and the objects were presented one on the top part and one on the bottom part of the screen.

To ensure that events did not unfold too quickly for dogs’ eye movements (Park et al. 2020), in our second experiment, we displayed slower actions. Moreover, to avoid the preference dogs showed for the upper part of the screen, we positioned the two objects on the right and left sides of the scene. Finally, to ascertain that the negative findings of Experiment 1 were not due to the low frame rate of the stimuli employed by Kano and Call (2014), we displayed videos at a higher frame rate in Experiment 2.

## Experiment 2 - methods

The experimental design, hypothesis, predictions, expected sample size and size of the target AoIs for the anticipatory looking analyses were pre-registered: https://osf.io/sm5gr.

### Subjects

We tested nineteen dogs (8 females) of different breeds. Eleven of these individuals participated in Experiment 1, which had taken place at least thirteen months earlier (compare ages between Tables S1 and S2; dogs with the same ID number between tables were tested in both experiments). The average age at the beginning of testing was approximately 60 ± 28 months. More details about the demographic and counterbalancing information are shown in Table S2. On average, dogs needed approx. 11 (± 5) training sessions.

### Stimuli

The stimuli used in this experiment are available at https://github.com/cvoelter/dog_woodward_2022/tree/main/stimuli_Exp_2. Videos were shown at a frame rate of 100 Hz and occupied 1024 × 431 px (32° x 13.5° of visual angle). They were presented contingent on the dogs’ gaze being detected for at least 200 ms in the centre of the screen to ensure that the dogs would start watching the first trial with their gaze centred between the two objects. The familiarization videos consisted of three trials (identical repetitions) of a 10 s recording in which the agent approached one of the two objects. The first and last frames of the familiarization videos were presented statically for 1 s. In the first frame, the agent was central between the two objects. Approximately 1 s after the beginning of the video, the agents provided a directional cue as to where they were going: the human agent turned her head and looked toward the object she was going to approach, and the inanimate agent started to move by orienting itself in the direction of the target object. The approach sequence lasted approximately 4 s, after which the agents made contact with the objects. The human agent kicked the object with her foot twice and the inanimate agent bumped into it twice. Both agents took a step back before contacting the object a second time. The videos were synchronized with regard to the frame in which the objects started to move after being touched by the agents for the first time.

After the three identical repetitions of the familiarization event, we showed for 5 s a static frame depicting only the two objects, with locations swapped relative to the previous trials. No agent was present in the scene during this last familiarization trial.

Between familiarization trials, a grey screen was shown for 1 s. After the last familiarization trial, the dogs were presented again with a central animation before the test trial was shown. The first frame of the test videos, depicting the agent centred between the two objects, was presented statically for 4 s. After that, the events of the test unfolded similarly to those in the familiarization, but the last frame was frozen and presented statically for 10 s.

We considered as AoIs squares, centred around the two objects, with a side of 230 px for all analyses concerning anticipatory looks and a side of 260 px (to encompass also the area where the objects would move after being pushed by the agents) for the analysis of the dwell times at the end of the videos.

### Design and procedure

**Fig. 3:**
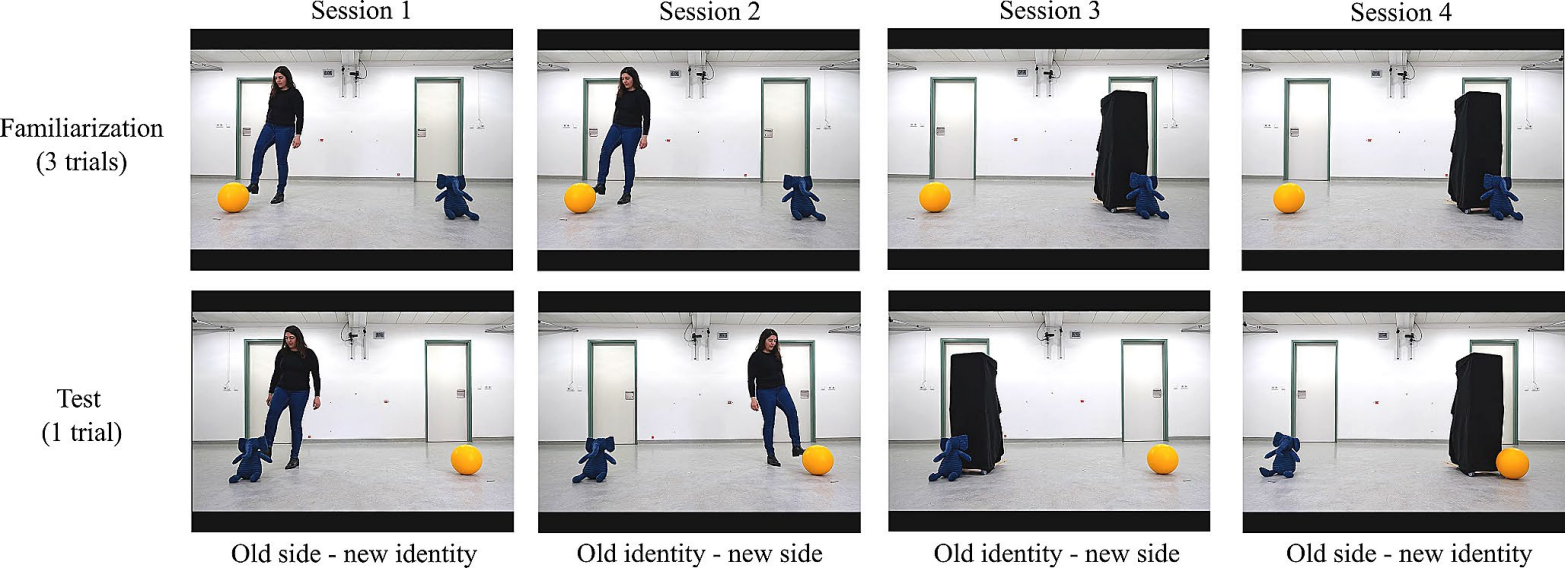
Experimental design. The order of presentation of the agents (human or inanimate sessions first), the target object the agent interacted with during familiarization of the first session (ball or elephant) as well as the order of the test conditions (old side or old identity first) were counterbalanced across dogs

In a 2 × 2 within-subject design, each dog was tested in four test trials, each depicting a different combination of agent and condition. Dogs were tested on four sessions that were separated by at least one week. In each session, the dogs were shown a full familiarization and a single test trial. We counterbalanced the agent (human or inanimate) and the condition (new identity or new side) shown in the first session across subjects. For the agent, we presented the sessions in a blocked (AABB or BBAA) order, counterbalanced across dogs. The side of the familiarization target object (left-right) and its identity (ball-elephant) were counterbalanced across dogs. Unlike in Experiment 1, for each dog, both objects served as targets during the familiarization, one object per agent. For example, if a dog saw the human agent approach the ball on the left side during familiarization, the inanimate agent for that dog would approach the elephant on the right side during the familiarization (Fig. 3). The data were collected with the same set-up as in Experiment 1 (Fig. 1a-b).

### Statistical analyses

#### First fixated object during the first four seconds of the test

The IP for this variable consisted of the first 4 s of the test video, when the agents were immobile (the human agent facing straight ahead) and centrally placed between the two objects. The first (frozen) frame of the test video was shown only when the dogs fixated for at least 200 ms on a centrally presented animation. This ensured that the dogs started the test trial with their gaze on the agent. We scored whether the dogs’ first fixation was directed to the old identity object (coded as 1) or to the old location object (coded as 0). We additionally coded as 0 s the instances in which dogs did not fixate into any of the objects AoIs during the IP in any of the trials. For this binary dependent variable we fitted a binomial model with a logit link function (McCullagh and Nelder 1989). We included the agent shown on the trial (human or inanimate) as test predictor and the session number within agent (1 or 2) and the order in which the sessions were presented to the dogs (human sessions first or inanimate sessions first) as control predictors. The random slope of agent within subject was also included, after being dummy coded and centred. Inference about the fixed effects was drawn using the function *drop1* with the argument “test” set to “Chisq”.

We additionally performed a McNemar test (considering only each dog’s first fixation to any of the objects, irrespective of whether this happened during the first or second trial) to assess whether the proportion of first fixations to the old identity or old location objects differed between the two agents.

Finally, we fitted a binomial, intercept-only model to test whether the dogs’ first fixations were directed at the old identity object significantly above chance level. We included as control predictors with fixed effect the session number (z-transformed) and the order of presentation of the sessions. We also included the random intercept of the subject.

#### Latency to make a first fixation to any of the objects before the agent moves (first 4 s of test)

We used an LMM to investigate whether the latency of the dogs to make a first fixation to one of the AoIs was influenced by the object in the AoI (new identity or new side object) and by the agent performing the action (human or inanimate). This measure was analysed for comparability with the developmental literature (e.g., Krogh-Jespersen and Woodward 2018) and because there is already evidence that dogs’ latency to look at agents’ immediate action targets is influenced by the species (e.g., dog vs. human) of the agent (Lonardo et al. 2023). As test predictor, we included the interaction between AoI and agent and their main effects because we wanted to investigate whether a potential difference in the latencies to make a first fixation to the two AoIs would depend on the agent performing the action. Subject was included as random intercept. We fitted the model using the function *lmer* of the package *lme4* (version 1.1.31; Bates et al. 2015).

#### Proportion of dwell time to the old identity object during the first 4 s of test

To analyse the proportion of dwell time to the old identity object (dwell time to old identity divided by total dwell time to the two objects) during the first 4 s of the test videos, we fitted a GLMM with beta error structure and logit link function (Bolker 2008; McCullagh and Nelder 1989), using the function *glmmTMB* of the homonymous package (version 1.1.5; Brooks et al. 2017). As sole test predictor, we included the type of agent (human or inanimate). We controlled for the session number within agent (1 or 2, z-transformed), the order of presentation of the sessions (human or inanimate sessions first) and the identity of the “old target” object (ball or elephant). We included the random slope of agent (manually dummy coded and centered) and of the z-transformed session number within agent within the random intercept of subject.

#### Pupil size during test events

Like in previous studies with dogs (Völter and Huber 2021, 2022; Völter et al. 2023), we analysed the pupil size during a 4 s IP (from 4001 to 8001 ms), when the agent started to move. For each subject and trial, we calculated the median pupil size during a baseline period, the second immediately preceding the IP (from 3000 to 4000 ms, when the agent was presented statically, centred between the two objects). We then subtracted the median pupil size during the baseline period from the interpolated data (only gaps of up to 500 ms were interpolated) for each dog and trial. Finally, we downsampled the pupillometry and gaze coordinates data to 10 Hz, by retaining the median value for each time bin, to reduce autocorrelation in the signal, potentially deriving from the relatively slow pupil dilation response compared to the high original (1000 Hz) sampling rate. More details are provided in the SM.

To the pre-processed and down-sampled data, we fitted a generalized additive mixed model (GAMM) with Gaussian error structure, using the bam function of the R package *mgcv* (version 1.8.41; Wood 2011), with the smoothing parameter selection method set to “ML”. We included linear terms for condition, agent and their interaction. To fit different smooths for the different levels of the categorical variables, we included the factor-smooth interaction between both agent and condition on the one hand and time on the other hand. This allows us to model the different effects of time depending on the level of the categorical variables (agent and condition). Indeed, we expected the effect of time on pupil size to be non-linear but not necessarily identical between levels of these categorical variables. We additionally included a smoothing term for the interaction between the X and Y gaze coordinates, as the gaze position on the screen might influence pupil size (Mathôt et al. 2018). Finally, we included a random smooth term for the interaction between time and each combination of subject, test event (human-new identity; human-new side; inanimate-new identity; inanimate-new side) and session number. All smooth terms had the maximum number of basis functions set to 20, to allow to fit more wiggly patterns (van Rij et al. 2019). We compared the fit of this full model with that of a null model lacking the linear interaction between agent and condition and the factor-smooth interactions between condition and agent and time, using the function *compareML* of the package *itsadug* (version 2.4.1; Van Rij et al. 2015). Additionally, to evaluate the significance of condition, we visually inspected the estimated differences between conditions using the function *plot_diff* of the same package.

## Experiment 2 - results

### First fixated object during the first 4 s of the test

Aggregating the data across dogs and trials, on average 8 (SD ± 27) % of the dogs fixated the old identity object first when the agent was human and 21 (SD ± 41) % when the agent was inanimate; 26 (SD ± 27) % of the dogs fixated on the old side object first when the agent was human and 21 (SD ± 41) % when the agent was inanimate. The remaining dogs did not make anticipatory fixations.

**Fig. 4:**
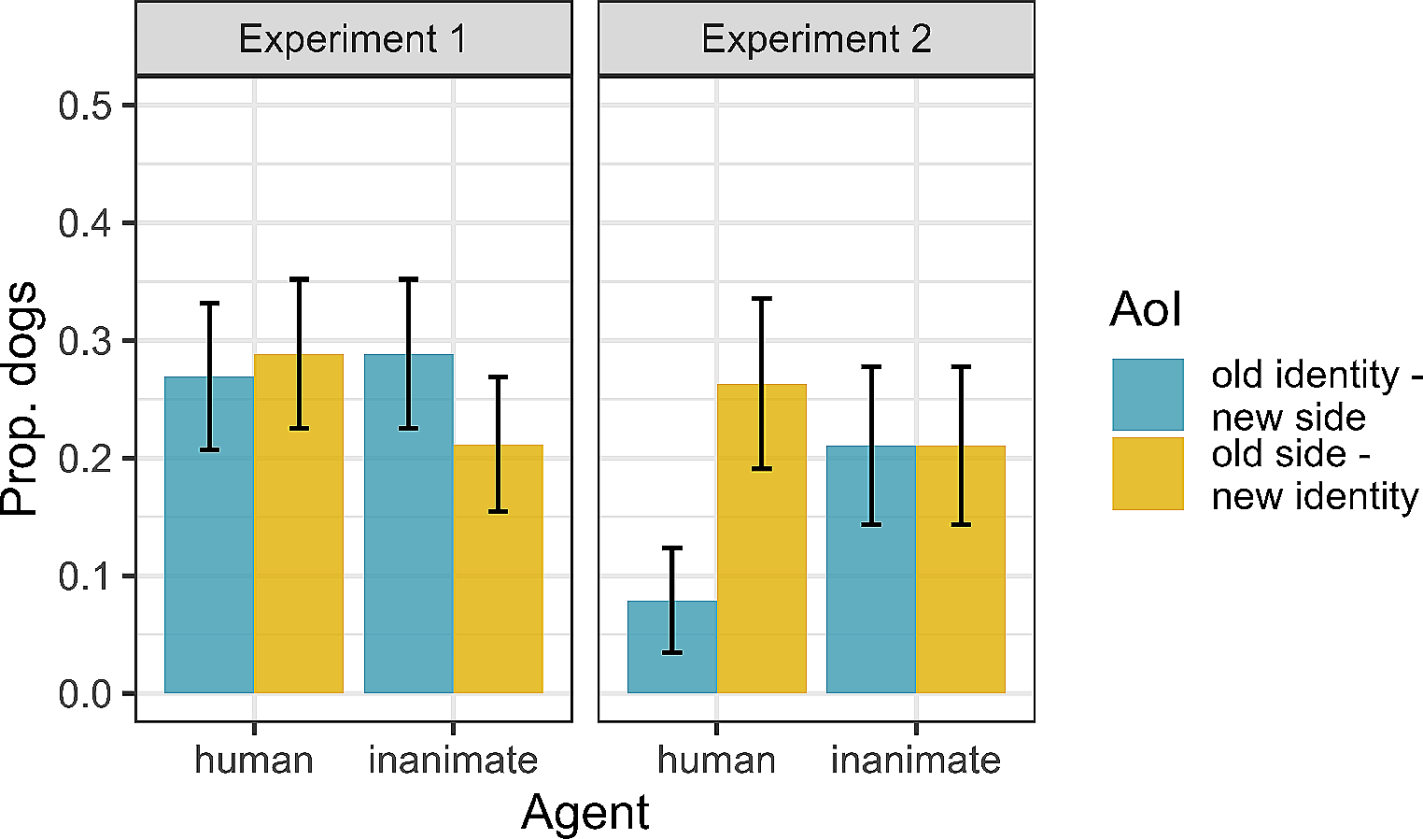
Mean proportion of dogs making their first anticipatory fixation to the old identity/new side or old side/new identity object over the four trials per agent in Experiment 1 (left panel) and over the two trials per agent in Experiment 2 (right panel). The remaining proportion of dogs did not make anticipatory fixations. Error bars represent standard errors

The results of the binary model (Table S11) indicate that the dogs’ probability of looking at the old identity object was not affected by whether the agent was human or inanimate (χ^2^_1_ = 1.44, *p* = .231). When the agent was human, three of the dogs that exhibited anticipatory looks during the IP fixated first on the old identity object and the remaining eight on the old side object. When the agent was inanimate, eight dogs fixated on the old identity object and eight fixated on the old side object first. A McNemar test with continuity correction indicated that these proportions were not significantly different from each other (χ^2^_1_ = 0.071, *p* = .789), thus confirming the results of the binary model that, when the dogs showed anticipatory looks at the objects, these first fixations were not systematically directed to the old identity or to the old side (Fig. 4). Similarly, the intercept-only model confirmed that dogs’ first fixations were not directed at the old identity object significantly above or below chance level (estimate of the intercept: -0.49 ± 0.4, Wald test: z = -1.24, *p* = .214). We additionally conducted a power analysis on this model (details in the SM). This was not based on the effect size observed in our study, which is a biased procedure (Dziak et al. 2020), but on the effect size reported by Cannon and Woodward (2012). The power analysis suggested that even for a large effect size, with our sample size the model we used was likely underpowered (Table S16). Given this result, we decided to test also for the absence of an effect, by conducting equivalence tests (Lakens et al. 2018). We used as the smallest effect size of interest the one obtained by Cannon and Woodward (2012) and found that the probability of looking at the old identity object was statistically equivalent between agents (two one-sided tests : t_31.14_ = 2.48, *p* = .009). This suggests that the probability to look at the old identity object in our study is not affected by the agent (assuming that the effect size found by Cannon and Woodward is the smallest effect size of interest).

### Latency to make a first fixation to any of the objects before the agent moves (first 4 s of test)

From the start of the IP, on average dogs took 1343 (± 652) ms to make their first fixation into the old identity/new side AoI and 1153 (± 1227) ms to make their first fixation into the old side/new identity AoI, when the agent was human. When the agent was inanimate, dogs’ average latency to make their first fixation into the old-identity/new-side AoI was 1596 (± 1371) ms and their average latency to make their first fixation into the old side/new identiy AoI was 910 (± 1080) ms.

**Fig. 5:**
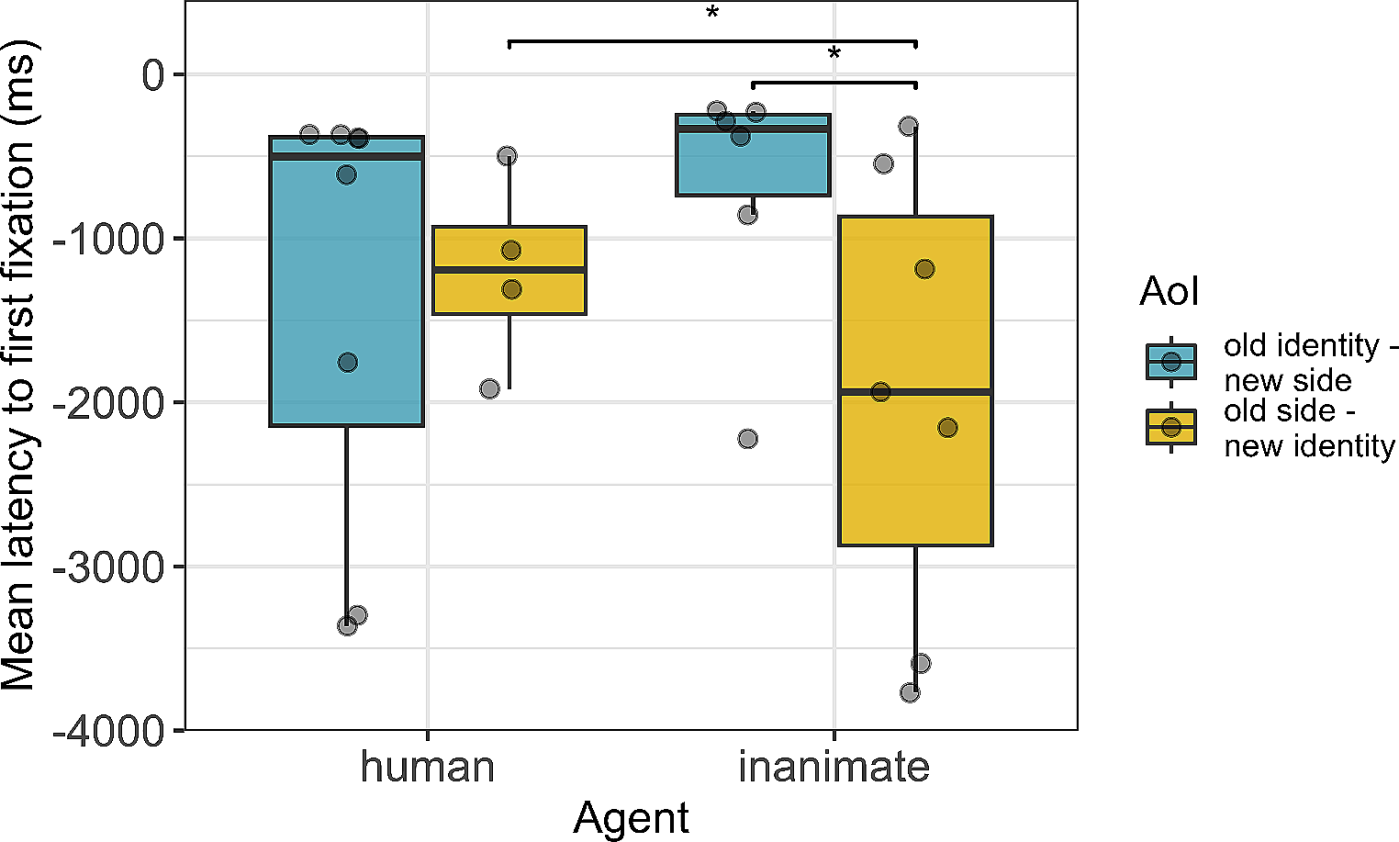
Experiment 2: Boxplots showing the average latency to make a first fixation to the old identity object (shown in blue) or old side object (show in yellow). The IP shown on the y-axis corresponds to the 4 s in which the first frame of the video was shown, before the agent started to move. Larger absolute values indicate faster latencies. Dots indicate individuals’ latencies. Asterisks indicate *p* < .05 (LMM)

Dogs’ fixated on the new identity/old side AoI faster when the agent was inanimate than when it was human (t = -2.18, df = 22.47, *p* = .041; Table S12 and Fig. 5). Furthermore, in the inanimate condition, they were slower at fixating the old identity/new side AoI than the old side/new identity AoI (Estimate = 1654.57 ms +/- 541.85 ms, df = 25.34, t = 3.05, *p* = .005; Fig. 5). This was not the case in the human condition, where dogs’ latency to make a first fixation did not differ between the two object AoIs (t_19.02_ = 0.12, *p* = .908; Table S12 and Fig. 5).

### Proportion of dwell time to the old identity object during the first 4 s of test

On average, when the agent was human, the dogs’ proportion of dwell time into the old identity/new side AoI was 0.11 (SD ± 0.3) and that into the old side/new identity AoI was 0.23 (SD ± 0.41). When the agent was inanimate, the proportion of dwell time into the old identity/new side AoI was 0.22 (SD ± 0.41) and that into the old side/new identity AoI was 0.21 (SD ± 0.41). On average, dogs looked equally long to the old identity/new side and to the old side/new identity object (exploratory paired-samples, two-sided t-test: t_18_ = -0.92, *p* = .372).

The type of agent (human or inanimate) did not affect dogs’ proportion of dwell time to the old identity object. This variable was not influenced by the session number, the order of presentation of the sessions, nor by the identity of the old identity object (ball or elephant), as reported in Table S13.

### Pupil size during test events

The full-null model comparison revealed that the full model explained the response better (the full model had lower AIC; Δ AIC: 32.58, difference of ML scores: χ^2^_11_ = 15.09, *p* = .001). Because the linear interaction was not significant, we removed it and retained only the main effects of condition and agent in the full model.

**Fig. 6:**
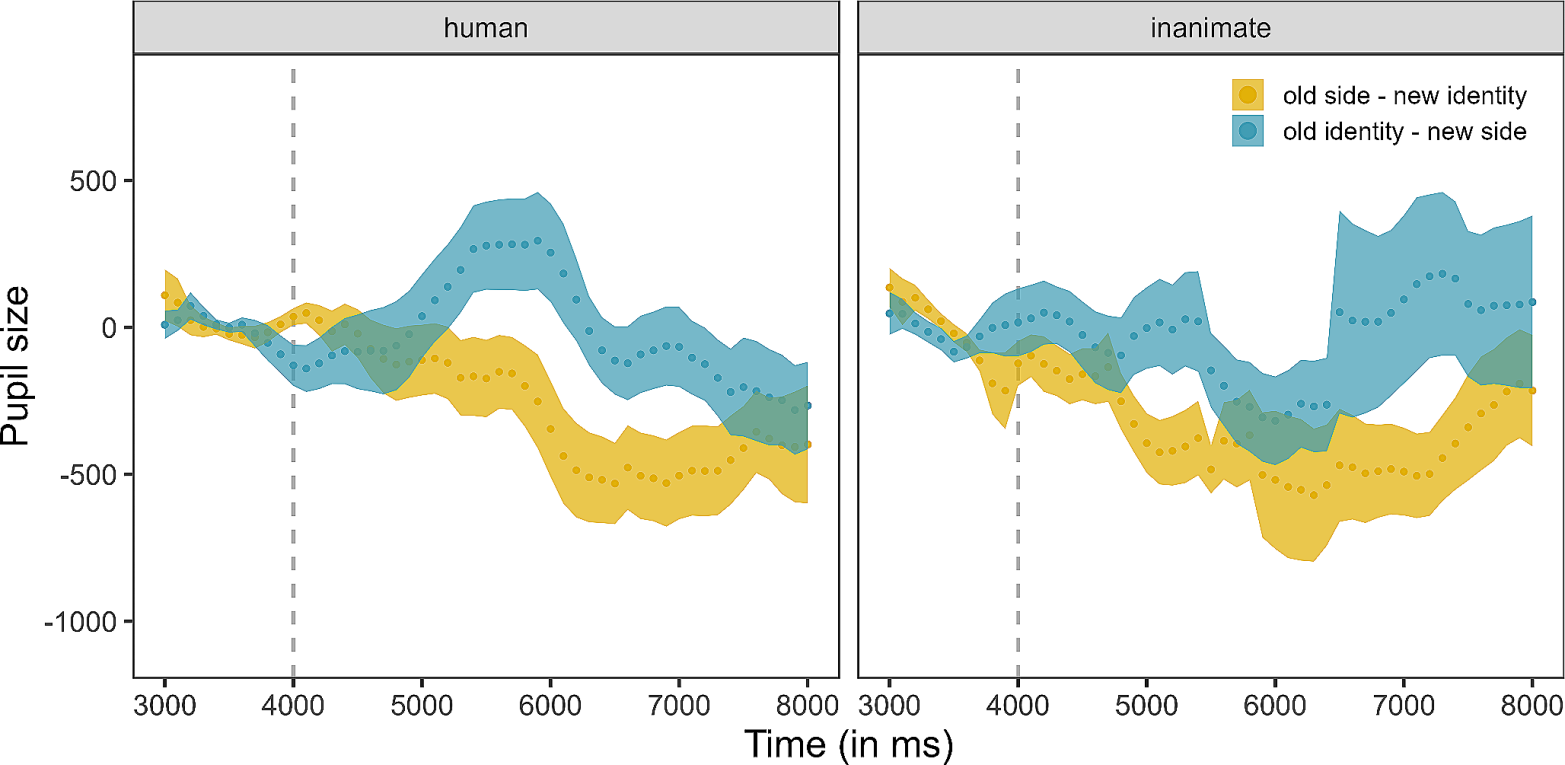
Experiment 2: baseline-corrected and downsampled pupil size in the two conditions, plotted separately for the human (panel on the left) and inanimate agent (on the right). The grey dashed line represents the end of the baseline period and each dot shows the mean pupil size value for a 10 Hz bin. The dotted lines and the coloured areas around them represent the means and their standard errors, respectively

In the old identity - new side condition, the dogs’ pupils were generally larger than in the old side/new identity condition, irrespective of the agent shown (t = 2.36, *p* = .019; Table S15, Fig. 6 and Fig. S4). The agent (human or inanimate) did not affect dogs’ pupil size (t = 0.47, *p* = .638). Finally, the gaze coordinates on the screen (F = 29.37, *p* < .001) and the combination of subject, session number and test event (F = 195.27, *p* < .001) significantly contributed to explaining the pupil size. The exclusion of two trials in which the dogs’ eye was not tracked for more than 30% of the trial duration (following previous work, e.g., Völter et al. 2020) did not affect the results.

## General discussion

The present study aimed to investigate which aspects of an agent’s goal-directed behaviour dogs perceive as relevant to the agent’s underlying intention. Specifically, we asked whether dogs preferentially expect the target object or the location where the action takes place to be the agent’s “goal” and whether dogs’ action perception is influenced by the animacy of the agent. Although the dogs’ first fixations and dwell times (see SM) did not reflect a preferential encoding of the identity over the location of the agents’ actions, the dogs had larger pupil sizes when the agents changed their movement trajectory than when they changed the identity of their target object.

In neither experiment did dogs predict systematically with their first fixations the old side or the old identity object. Thus, their anticipatory looks in both tasks resembled those of 8-month-old infants who did not receive a first-person grasping training (Krogh-Jespersen and Woodward 2018). Testing whether dogs’ predictive gaze shifts can be influenced by first-person training on the observed actions constitutes an interesting avenue for future research. However, an important difference between our findings with dogs and those of previous studies with infants is that dogs did not even show online anticipatory fixations during familiarization trials. The small sample size and the low number of dogs that exhibited anticipatory fixations during test trials prevented conclusions being drawn from these measurements. For example, in Experiment 2, 11 dogs made an anticipatory fixation on one of the objects: three looked first at the old identity object and eight looked first at the old location object, when the agent was human.

Similarly to what was found in human infants (Krogh-Jespersen and Woodward 2018), dogs looked earlier at the old side than at the old identity object, although this difference reached statistical significance only for the inanimate but not the human agent. Dogs might expect an inanimate but not a human agent to follow along the same trajectory. However, the pupil size results seem to suggest that dogs found the new side event more surprising than the new identity one, irrespective of the animacy of the agent. Another possibility to explain the difference between agents in dogs’ latency to make a first fixation is that dogs expected both agents to continue following the old side trajectory but their gaze arrival times to the objects were slower when the agent was human, due to the increased saliency of a human compared to an inanimate agent. Therefore, slower latencies to the old side object when the agent was human than when it was inanimate might not reflect a reduced expectation but rather more interest in the human agent. As previously hypothesized (Lonardo et al. 2023), the more salient an agent is, the more difficult it is for dogs to find to visually disengage from it and hence the slower it is to look at the agent’s action target. Consequently, the difference in the latency to make anticipatory looks at the old identity and old side object is best interpreted within agent. For this reason, in this study, we intentionally refrained from presenting dogs with a conspecific agent. Although observing a conspecific executing a familiar action theoretically ensures motor representations of the movements for the observers, previous evidence (Lonardo et al. 2023) suggests that effector-specific movement representations of the observed actions are not required for dogs to visually anticipate others’ action targets. Moreover, the heightened saliency of a conspecific presence has raised concerns about potential disruptions to anticipatory gazes in dogs (Lonardo et al. 2023). Nevertheless, displaying conspecific agents in future studies using Woodward’s paradigm would be a valuable addition to the current literature.

In Experiment 2, dogs’ dwell time was longer for the human than for the inanimate agent, supporting the hypothesis that they find human agents more salient than inanimate, albeit self-propelled, ones. These results are in line with a previous study in which dogs fixated longer on pictures of human faces than on pictures of inanimate objects (Somppi et al. 2012). It might also be that dogs lack motor experience with kicking actions (as performed by the human agent in our study) but the experience of “bumping into” an object (as the inanimate agent did) might be more familiar to them.

Because in our second experiment, the dogs saw the completion of the action, they received “feedback” on their prediction, which could have potentially influenced their prediction in the second trial involving the same agent. However, sessions were presented at least one week apart, decreasing the risk of carry-over effects, and we controlled for the effect of trial number in the analysis. The results showed that indeed there was no effect of trial number, making it unlikely that observing the completed action in the first trial systematically influenced dogs’ predictions in the second trial with the same agent.

In the second experiment, dogs looked equally long when the agents approached the old identity object or the new object on the old side, similarly to pre-reaching, 3-month-old infants (Woo et al. 2023). Interestingly, using the violation of expectation paradigm, Woo and colleagues found that pre-reaching 3-month-old infants can flexibly form expectations about what constitutes the “goal” of a human agent’s reach, as long as more explicit behavioural cues than those shown in our study are present. Testing whether a similar approach (an agent repeatedly reaching for a location irrespective of the object there or repeatedly reaching for an object irrespective of its location) could influence dogs’ looking times in this paradigm is another question for future research.

During the familiarization trials of both experiments, overall dogs did not predict with their gaze the target of the action, using the trajectory of the agents’ movements. Therefore, we included all dogs in the analyses of test trials, unlike previous research that only analysed the performance of infants who predicted the action outcome by the end of the familiarization phase (e.g., Southgate et al. 2007). Our null findings concerning anticipatory fixations could be due either to a lack of systematic expectation that agents will approach the old location/object in dogs or to the anticipatory looking paradigm (with only 3 familiarization trials, as in Cannon and Woodward 2012) being unsuitable for testing dogs’ processing of goal-directed actions. This latter possibility is supported by the rarity of online anticipatory fixations during familiarization as well as test trials. Specifically, it might be that the events, especially in Experiment 1, unfolded too quickly for dogs or that dogs had difficulties associating the agent’s actions with their target with only limited exposure (three trials). However, the pupil size results of Experiment 2 seem to reflect at least an implicit association between the agents and their previous trajectory or preferred location. Moreover, from the latencies of dogs’ first looks at the objects during the first familiarization trials of both experiments (see SM), we can conclude that dogs paid attention to the objects, and some gazed at them predictively as the action was still unfolding but on average gazing at the objects when the agents made contact with them. Hence, a general lack of interest/attention toward the stimuli cannot explain why dogs, unlike humans and apes, did not show systematic anticipation of one object over the other before the agents started to move during test trials. While it is possible that dogs lack expectations about the observed actions, also differences between the visual systems of dogs and primates might explain our results. The relatively scarce data we have (Lonardo et al. 2023; Park et al. 2020) on the matter hints at the possibility that dogs’ (slower) scanning patterns to object-directed actions might not be directly comparable to those of humans and apes. Indeed, from the studies mentioned above it seems plausible that dogs do not show as frequently as humans and apes anticipatory fixations to the target objects of others’ actions. In particular, it is possible that dogs’ visual streak, and hence increased visual acuity along the periphery of their visual field (Barber et al. 2020), renders anticipatory looks to action targets less relevant than for primates. Until more research has tested this hypothesis (e.g. by comparing breeds with more or less pronounced visual streak; McGreevy et al. 2004), conclusions drawn based on the prevalence of anticipatory looking in dogs should be interpreted with caution.

Nevertheless, other measures can tap into dogs’ processing of goal-directed actions. For example, when averaging dogs’ looking times across three test trials, Marshall-Pescini and colleagues (2014) find a significant difference between conditions. The lack of difference we found between looking times to the old side/new identity and old identity/new side test event resembles the results of Marshall-Pescini et al. (2014) who, in the analysis of their first trial only, did not find a difference in the looking times between conditions. We only presented dogs with four (Experiment 1) and two (Experiment 2) test trials per agent due to limitations in our dogs’ availability and because we wanted to avoid losing the dogs’ interest due to repetitive stimuli. Indeed, in a previous eye-tracking study (Lonardo et al. 2023), dogs’ looking time decreased with increasing trial number over the course of four identical trials within the same session. Moreover, the predictive gaze-shifts infants showed in the study by Cannon and Woodward (2012) were evident already from the first test trial. In addition to having presented fewer test trials per condition, we presented, on separate days, the human and inanimate agents within subject, unlike the between-subjects designs employed by the Marshall-Pescini (2014) and Woodward (1998)’s studies. An additional difference between our study and those by Marshall-Pescini and colleagues (2014) and Woodward (1998) is that we did not habituate the subjects to the events but rather presented a fixed number (three) of familiarization trials. The median number of trials dogs needed to habituate in Marshall-Pescini et al.’s study was nine and Woodward (1998) presented infants with at least six trials during habituation. Finally, our human agent kicked the objects using her feet, instead of crouching down and touching the objects with her hands. Therefore, it is possible that dogs require more visual experience and potentially habituation to the action shown, in order to exhibit a difference in looking times between the new side and new identity events. This would be consistent with the finding in human infants that looking times actually measure the learning happening during the habituation phase of an experiment rather than previous knowledge they might already possess (Jackson and Sirois 2022). The adequacy of three familiarization trials in enabling dogs to encode the target object’s identity as the agent’s “goal” remains uncertain. An increasing number of familiarization trials could alter dogs’ perception, potentially leading to prolonged dwell times in response to the event involving a new identity at the old location. Intriguingly, it is noteworthy that a mere three familiarization trials proved sufficient for human infants and great apes to elicit anticipatory looks to the old-identity object. This suggests they could encode a human manual action as being directed at a specific object after only a brief exposure (Cannon and Woodward 2012; Kano and Call 2014). Furthermore, the same number of familiarization trials yielded discernible differences in dogs’ pupil sizes between conditions. This effect manifested during the video segment in which the agents started to move until just before contact with the objects. Remarkably, during this interval, dogs reacted more to the agents changing their path rather than the identity of their target object, relative to familiarization. Hence, three familiarization trials proved adequate for dogs to encode the location of the action.

When analysing the pupil size data, we controlled for luminosity of the stimuli in two ways. First, we included in the GAMM the interaction between X and Y gaze coordinates, to control for the effect of eye orientation and region of the video looked at on pupil size. Even after controlling for this, the effect of condition on pupil size remained significant. Second, given our counterbalanced design, the exact same test video (e.g., human approaching ball on the left side) represented for some dogs the “old side” event and for other dogs the “old identity” event.

The pupillometry results suggest that dogs responded more to the perceptual novelty (a change in the agent’s path) of the old identity/new side event compared to the conceptual novelty (a change in the identity of the agent’s target object) of the old side/new identity event. The fact that dogs’ pupil size reflected an expectation that the agents should have kept approaching the same side is consistent with evidence from previous studies showing how locations might be easier to encode for dogs (Fugazza et al. 2016; Piotti et al. 2018). For example, Piotti and colleagues found that, in a reversal learning task, dogs trained to respond to the location of the stimuli learned faster than those trained on the characteristics of the stimuli. However, why the pupil size, but not the dwell times, reflected a location-based expectation warrants further investigation. Concerning the anticipatory looks, descriptively dogs were more likely to make their first fixation to the old side object, but this result did not reach significance, possibly because our model was underpowered to detect an effect (see power analysis in SM).

In sum, our findings do not support the hypothesis that dogs (Marshall-Pescini et al. 2014), similarly to human infants (Cannon and Woodward 2012; Woodward 1998) and apes (Kano and Call 2014), preferentially encode the identity of others’ goal-directed action targets. Instead, the pupil size results provide some evidence that dogs might preferentially encode the spatial location where the action takes place, irrespective of the animacy of the agent. It is possible that dogs, similarly to younger infants before they have had sufficient motor experience, did not visually anticipate the outcome of the actions because they lack motor representations for the observed movements and the agent’s goal (the object or its location) was not clearly disambiguated during familiarization (Krogh-Jespersen and Woodward 2018; Sommerville et al. 2005; Woo et al. 2023). Finally, dogs looked longer to a human than to an inanimate, albeit self-propelled, agent, possibly hinting at the relevance of faces and biological motion.

## Electronic supplementary material

Below is the link to the electronic supplementary material.

Supplementary Material 1

## Data Availability

The data, R scripts and supplementary materials associated with this manuscript are available on GitHub: https://github.com/cvoelter/dog_woodward_2022.
