# Supplementary material for: Do dogs preferentially encode the identity of the target object or the location of others’ actions?
Source: Anim Cogn. 2024 Mar 30;27(1):28. doi: 10.1007/s10071-024-01870-w (PMC10980658; doi:10.1007/s10071-024-01870-w)
Supplement: Supplementary file 1 — Supplementary Material 1 [file 10071_2024_1870_MOESM1_ESM.docx]

Supplementary materials for: Do dogs preferentially encode the identity of the target object or the location of others’ actions?

Animal Cognition

Lucrezia Lonardo, Christoph J. Völter, Robert Hepach, Claus Lamm and Ludwig Huber

Corresponding author: Lucrezia Lonardo, lucrezia.lonardo@vetmeduni.ac.at; Comparative Cognition, Messerli Research Institute, University of Veterinary Medicine of Vienna, Medical University of Vienna and University of Vienna, 1210 Vienna, Austria.

Supplementary text

# **Statistical analyses**

## Experiment 1

**Latency of first look to the objects during familiarization.**

In both experiments, following Krogh-Jespersen & Woodward (2018), during the first trial of each familiarization session, we measured the dogs’ action prediction, operationalized as their gaze arrival time to both objects (target, i.e., the object approached by the agent, and distractor, i.e., the ignored object) relative to the moment in which the agents made contact with the target. We were interested in a possible effect of the agent on this latency as the differing motion/kinematic cues of the two agents or the differing familiarity the dogs had with the two types of agent might have influenced their ability to predict their target object based on their motion alone.

In Experiment 1, the interest period (IP) for this analysis consisted of the first trial of each familiarization session. We measured dogs’ gaze arrival time to the objects relative to the moment in which the agent in the video made contact with her target. We define as “target” the object the agent reaches for, while as “distractor” the other, ignored object. Because we subtracted from the gaze arrival time the time when the agent made contact with the object, positive values of this variable indicate reactive looks and negative values indicate predictive looks (i.e., looks to the object happening before the agent had made contact with it). To ensure we only measured anticipatory looks based on the agents’ motion, we excluded from this analysis gaze samples falling into the objects AoIs that were not preceded by looks to the agent. In order to retain fixations only, we additionally excluded gaze samples that were categorized as saccades (i.e., with an acceleration faster than 30° of visual angle/s) or blinks. We ran two distinct LMMs (Baayen, 2008), one for the gaze arrival times to the target object and one for the gaze arrival times to the distractor, with the same structure and predictors. Namely, we tested whether the gaze arrival times were influenced by the agent (human or inanimate), as only test predictor, and by the agent shown first, the session number (1 to 4, z-transformed) and the location of the target object (upper or lower part of the screen) as control predictors. We additionally included the random slope of session number within subject and a parameter for their correlation.

We used two-tailed, one-sample t-tests to assess whether dogs’ first looks to the objects were significantly predictive or reactive relative to the moment in which the agent made contact with them. We adjusted the *p*-values for multiple comparisons using the Holm-correction.

### Proportion of dwell time to the old-identity object

The model was somewhat overdispersed (dispersion parameter: 1.26). The distribution of the best linear unbiased predictors (BLUPs; Harrison et al., 2017) was roughly symmetrical. We assessed that collinearity among the predictors was not present (all VIFs close to 1). The model was fairly stable.

### First fixation

The distributions of the Best Linear Unbiased Predictors (BLUPs) were roughly symmetrical.

## Experiment 2

### Latency of first look to the objects during familiarization

This was was analysed as described above (section “Statistical analyses - Experiment 1 – latency of first look to the objects during familiarization”), with the exception that we additionally excluded from this analysis looks to the objects that happened before the agent started to move. Indeed, unlike in the stimuli of Experiment 1, in the stimuli of Experiment 2 the agents only started to move approx. 1.6 s into the video.

Using two-sided, one-sample t-tests on aggregated data, we tested whether dogs’ gaze arrival times to the objects were significantly different from 0 (i.e., the moment in which the agent in the video contacted the objects). Dogs gazed at the target of the inanimate agent on average 1287 (SD ± 2856) ms before this moment and at the distractor 359 (SD ± 3433) ms in advance. When the agent was human, dogs looked at the target 214 (SD ±1916) ms and at the distractor 2298 (SD ± 2553) ms before the moment in which the agents made contact with the objects. After adjusting the p-values for multiple testing using the Holm correction, none of the values resulted to be significantly different from zero. Only seven dogs looked at the distractor while 15 looked at the target at least once across the two trials when the agent was human. Twelve dogs looked at the distractor and all 19 looked at the target at least once across the two trials when the agent was inanimate.

The correlation between random slope and intercept was included in the target looks model, but not in the distractor looks model because in the latter the number of observations was too small for the number of random effects. The random slope of agent within subject was not identifiable as only three dogs looked at the target in both trials showing each agent and none of them looked at the distractor in both trials of each agent.

The assumptions of normally distributed and homogeneous residuals were evaluated by visual inspection of a QQ-plot (Field, 2005) of residuals and residuals plotted against fitted values (Quinn & Keough, 2002), which did not suggest any obvious violation of the assumptions. The assumption of normally distributed BLUPs was met as well. For all models we assessed that collinearity was not present among test predictors in linear models, using the function *vif* of the package *car* (version 3.1.1; Fox & Weisberg, 2011). The stability of the estimates of all models reported in this study was assessed excluding one level of the grouping factor (i.e., most often one subject) at a time and fitting the model again (Nieuwenhuis, 2012). The effect of the test predictor for this model was stable even when excluding one subject at a time. For all models reported in the study, we calculated confidence intervals for the model estimates by means of a parametric bootstrap, using the function *bootMer* of the package *lme4* (version 1.1.31; Bates et al., 2015).

### First fixated object during first four seconds of test

The correlation between random intercept and slope was removed due to a convergence warning. For the same reason, it was not possible to include the random slope of session number within subject. Because we were only interested in the effect of the agent, we did not conduct a full-null model comparison. We ensured that the model was not affected by collinearity among predictors using the function *vif* of the package *car*. The fixed effects were stable.

### Latency to make a first fixation to any of the objects before the agent moves (first 4 seconds of test).

No obvious violation of the assumptions was detected. The assumption of normally distributed BLUPs was met as well. Collinearity was assessed for a model lacking the interaction (and the random intercept of subject) and revealed to be no issue (maximum variance inflation factor: 1.04).

Inference concerning the fixed effect of the interaction was drawn using the Satterthwaite approximation (Luke, 2017) using the function *lmer* of the package *lmerTest* (version 3.1.3, Kuznetsova et al., 2017) for a model fitted with restricted maximum likelihood. To obtain all pairwise comparisons, we relevelled the reference category of the factors in the model. The model stability and confidence intervals were calculated as described above.

### Proportion of dwell time to the old identity object during the first four seconds of test.

The correlation between random slopes and intercept as well as the random slope of the old identity object identity were not included due to convergence issues. Prior to fitting the model, we transformed the response variable so that it did not contain the exact values of zero and one (Smithson & Verkuilen, 2006). The model was based on 58 observations from 14 dogs (not all dogs looked to at least one object in all trials and five dogs never looked at the objects during the first four seconds of the videos). The model was not overdispersed (dispersion parameter: 0.99). Collinearity among test predictors, assessed for a linear mixed model, did not constitute a problem (all VIFs=1). The significance of the individual fixed effects was assessed using the function *drop1*, as described above. Confidence intervals and model stability were obtained as described above.

### Dwell time to the agents and their target object

This variable refers to the part of the video when the agents have already started to move. Specifically, the IP for this variable went from the moment in which the agent gave a directional cue towards her future target object/location until the end of the video (approx. 17 s). Before this IP started, the human agent had turned her head in the direction she was going to walk, while the inanimate agent had started moving in that direction.

To analyse the dwell times to the moving agents and their target object, we fitted a LMM (Baayen, 2008) using the function *lmer* of the package *lme4* (version 1.1.31; Bates et al., 2015). The dependent variable was obtained by summing up the total duration of fixations into the AoI encompassing the agent and the AoI encompassing the object upon which the agent acted. The sole test predictor was the interaction between condition (old side/new identity or old identity/new side) and agent (human or inanimate). We additionally controlled for a potential effect of session number (1 to 4, z-transformed) and of the order of presentation of the sessions (human or inanimate sessions first) by including these terms as fixed effects. We included the random slope of condition, agent and session number within subject.

Tests of the individual fixed effects were conducted using the Satterthwaite approximation for a model based on REML (Luke, 2017) with the function *lmer* of the package *lmerTest*. Because the interaction was not significant, we removed it and only retained the main effects of condition and agent in the full model. We then conducted a full-null model comparison (the null model lacked the effects of condition and agent), using a likelihood ratio test.

The factors were manually dummy coded and centered before entering the random slope part of the model. Correlation parameters between random slopes and intercept were not included due to a convergence issue. The model was based on all 76 observations (four trials per each of the dogs). We did not include in this model the identity of the target object so as not to increase model complexity given that we already had a low number of observations (5.9) per estimated term.

We visually confirmed that the assumptions of normality and homogeneity of the residuals were met and that the BLUPs were normally distributed. Collinearity among predictors, evaluated for a model lacking the interaction, was not present. The model was stable with regards to the fixed effects.

### Pupil size during test events

Samples from the 100 ms preceding and following each blink were removed to decrease the likelihood of artefacts in the signal. We subsequently filled the resulting gaps of up to 500 ms by means of linear interpolation. In two test trials (of two different dogs) the eye could not be tracked for more than 30% of the trial duration. In these trials, the eye was only tracked for 46% and 64% of the trial duration, due to one of the dogs’ third eye lid and due to the other dog’s extreme eye position when looking off-screen for some seconds, even without leaving the chinrest. Even including these two trials, on average, in the test trials, the dogs’ eye was detected for 94% of the trial duration and their gaze was on-screen (within the region of presentation of the stimuli) for 91% of the trial duration.

We assessed autocorrelation by visually inspecting the correlations between the residuals and the lagged residuals (function *acf* of the package *stats*; R Core Team (2021)). We checked the normality and homogeneity of the distribution of the residuals by inspecting a QQ-plot and a plot of residuals against fitted values (function *gam.check*, package *mgcv*). These checks confirmed that the autocorrelation was not excessive and that the assumptions of the model were met.

# **Results**

## Experiment 1

**Latency of first look to the objects during familiarization**

The latency of dogs’ first look to the target or the distractor AoIs did not depend on the agent, nor was it significantly influenced by the control predictors (Table S3 and S4). Dogs’ latencies to make a first look to one of the objects did not differ between the two object AoIs (difference between lower and upper AoI: t_12_= -1.53, *p* = .152).

Dogs’ first looks to the distractor were significantly predictive (i.e., they arrived at the distractor sooner than the agent arrived to the target) only when the agent was the inanimate claw (t_5_ = -7.46, *p _Holm_* = .003). The other gaze arrival times (to the distractor when the agent was a hand and to the target both when the agent was a hand and a mechanical claw) were not significantly different from zero.

## Experiment 2

### Latency of first look to the objects during familiarization

The dogs were approx. 1 s faster in looking at the target object when the moving agent was inanimate as compared to human (LMM: t_24.32_ = -2.07, *p* = .049; Table S9 and Fig. S2). The latency to look at the distractor object, instead, did not depend on the agent shown (LMM: t_22.25_ = 1.32, *p* = .199; Table S10). The agent shown in the first sessions as well as the session number within agent did not affect the latency to look at the target nor at the distractor.

Additionally, during the first trial of each familiarization session, we found no significant difference between dogs’ average looking time to the objects (paired samples, two-tailed t-test: t_18_ = 1.37, *p* = .189), suggesting that the dogs did not find one of the objects (ball or elephant) more salient.

**Dwell times to the agents and their target object during test**

The full-null model comparison reached significance (χ^2^_2_= 7.16, *p* = .028). Irrespective of condition, dogs looked longer at the human than at the inanimate agent (t_17.32_= -2.97, *p* = .009; Table S14, Fig. S3). The average dwell time to the agent/object was 9757 (SD ± 4741) ms when the agent was human and 6663 (SD ± 4626) when the agent was inanimate.

Dogs looked equally long to the agent/object between conditions (t_23.79_= -0.56, *p* = .583, Fig. S3). None of the control predictors explained the response significantly (Table S14).

### Power analysis for the binary model analysing the first anticipatory fixation during test

We simulated 1000 datasets replicating the design of Experiment 2: 76 observations, two per agent for each of 19 individuals. The agent shown first was balanced as in our collected sample (ten dogs starting with the human agent and nine with the inanimate agent).

We simulated the proportion of first fixations to the old identity object with the human agent to be 0.51 and 0.65 and with the inanimate agent 0.15 and 0.29. The highest values were exactly those observed by Cannon & Woodward (2012), while the lower ones were adapted from their study given that in our experiment we coded as 0s the trials in which dogs did not look at any of the objects. We assigned to the random intercept of subject the value 0.9 and to the random slope of condition within subject the values of 1.5 and 3. We chose these values because we expected the overall variation among dogs with regards to their probability to look at the old identity object in the two conditions to be large. We did not simulate any effect for the order of presentation of the agents and the session number. To each dataset we fitted the binomial model described in the section “Experiment 2 – Methods – First fixated object during first four seconds of test” to test the effect of agent on the probability to look first at the old identity – new side object.

With a simulated random slope of condition of 1.5, 17 of the 1000 models did not converge. With a 0.36 difference in performance between conditions, the model had a power of 76% and 69% (depending on the absolute values of the simulated performance) when the random slope of condition within subject was 1.5. The power was instead 47% and 43% when the random slope of condition was 3 (Table S16).

Tables

Table S1

Experiment 1: demographic information of the 13 tested dogs

| Dog ID | Sex | Neutered | Age (Months) | Breed | 1^st^ fam. agent and target |
| --- | --- | --- | --- | --- | --- |
| 01 | M | No | 24 | Collie | claw_frog |
| 02 | F | Yes | 40 | Labrador retriever | hand_frog |
| 03 | M | Yes | 27 | Mix | claw_duck |
| 04 | F | Yes | 32 | Labrador retriever | hand_duck |
| 05 | F | No | 27 | Mix | claw_frog |
| 06 | F | Yes | 44 | Mix | claw_duck |
| 07 | M | Yes | 65 | Mix | hand_duck |
| 08 | F | No | 31 | Border collie | hand_frog |
| 09 | M | No | 56 | Australian shepherd | hand_frog |
| 10 | M | Yes | 16 | Small Muensterlaender | claw_duck |
| 11 | M | Yes | 26 | Border collie |  |
| 12 | M | Yes | 24 | Mix | hand _frog |
| 13 | M | No | 63 | Border collie | claw_frog |

Table S2

Experiment 2: demographic information of the 19 tested dogs.

| Dog ID | Sex | Neutered | Age (Months) | Breed | 1^st^ fam. video and target | First condition |
| --- | --- | --- | --- | --- | --- | --- |
| 01 | M | No | 38 | Collie | Inanimate_ball_right | New side |
| 02 | F | Yes | 53 | Labrador retriever | Human_elephant_left | New side |
| 03 | M | Yes | 40 | Mix | Human_ball_right | New identity |
| 04 | F | Yes | 47 | Labrador retriever | Inanimate_ball_left | New identity |
| 05 | F | No | 42 | Mix | Human_elephant_right | New side |
| 06 | F | Yes | 60 | Mix | Inanimate_elephant_right | New identity |
| 07 | M | Yes | 79 | Mix | Inanimate_elephant_left | New side |
| 08 | F | No | 44 | Border collie | Human_ball_left | New side |
| 09 | M | No | 70 | Australian Shepherd | Inanimate_ball_right | New identity |
| 10 | M | Yes | 32 | Small Muensterlaender | Human_ball_right | New side |
| 11 | M | Yes | 40 | Border collie | Human_elephant_left | New identity |
| 14 | M | Yes | 69 | Australian shepherd | Inanimate_ball_left | New side |
| 15 | F | No | 79 | Flat coated retriever | Inanimate_elephant_left | New identity |
| 16 | M | Yes | 97 | Canarian warren hound | Inanimate_ball_right | New identity |
| 17 | F | No | 147 | Border collie | Inanimate_ball_left | New side |
| 18 | M | Yes | 87 | Mix | Human_elephant_right | New identity |
| 19 | F | No | 48 | Mix | Human_elephant_left | New side |
| 20 | M | No | 39 | Australian shepherd | Human_ball_right | New identity |
| 21 | M | Yes | 39 | Mix | Human_ball_left | New side |

Table S3

| Term | Estimate | Std. Error | t | Df | CI min | CI max | P | Min | Max |
| --- | --- | --- | --- | --- | --- | --- | --- | --- | --- |
| Intercept | -199.22 | 174.56 | ^(1)^ | ^(1)^ | -552 | 188.85 | ^(1)^ | -328.3 | -67.08 |
| Agent^(2)^ | 77.69 | 143.88 | 0.62 | 32.16 | -186.8 | 341.9 | 0.54 | -75.25 | 232.23 |
| Session number^(3)^ | -123.4 | 76.66 | -1.43 | 13.7 | -289.49 | 31.74 | 0.175 | -174.54 | -92.06 |
| Order^(4)^ | -311.29 | 204.21 | -1.4 | 8.27 | -734.74 | 76.36 | 0.197 | -629.7 | 62.07 |
| Location of target  ^(5)^ | 84.02 | 209.82 | 0.38 | 8.27 | -358.83 | 528.68 | 0.714 | -328.3 | -67.08 |

Experiment 1: results of the LMM investigating the effect of the agent (hand or claw) on the gaze arrival times to the target object during the first trials of familiarization.

(1) Not indicated due to its limited interpretability

(2) Reference category: claw

(3) Z-transformed to a mean of 0 and a standard deviation of 1

(4) Reference category: claw first

(5) Reference category: lower part of the screen

Table S4

Experiment 1: results of the LMM investigating the effect of the agent (hand or claw) on the gaze arrival times to the distractor object during the first trials of familiarization.

| Term | Estimate | Std. Error | t | Df | CI min | CI max | P | Min | Max |
| --- | --- | --- | --- | --- | --- | --- | --- | --- | --- |
| Intercept | -552.95 | 200.34 | ^(1)^ | ^(1)^ | -938.8 | -148.34 | ^(1)^ | -687.6 | -325.75 |
| Agent^(2)^ | 221.69 | 165.77 | 1.15 | 19.05 | -107.05 | 553.99 | 0.263 | 138.23 | 310.69 |
| Session number^(3)^ | -71.15 | 88.65 | -0.81 | 10.74 | -244.18 | 112.4 | 0.437 | -120.79 | 16.58 |
| Order^(4)^ | 110.32 | 230.59 | 0.48 | 7.26 | -377.48 | 578.64 | 0.644 | -195.65 | 270.12 |
| Location of target  ^(5)^ | -195.62 | 218.21 | -0.93 | 6.16 | -692.71 | 263.17 | 0.388 | -343.89 | 56.24 |

(1) Not indicated due to its limited interpretability

(2) Reference category: claw

(3) Z-transformed to a mean of 0 and a standard deviation of 1

(4) Reference category: claw first

(5) Reference category: lower part of the screen

Table S5

Experiment 1: results of the beta GLMM investigating the effect of the agent (hand or claw) on the proportion of dwell time to the old identity/new side object at the end of the test videos.

| Term | Estimate | Std. Error | χ^2^ | Df | CI min | CI max | P | Min | Max |
| --- | --- | --- | --- | --- | --- | --- | --- | --- | --- |
| Intercept | -0.16 | 0.27 | ^(1)^ | ^(1)^ | -0.69 | 0.42 | ^(1)^ | -0.39 | 0.17 |
| Agent^(2)^ | -0.09 | 0.26 | 0.14 | 1 | -0.61 | 0.4 | 0.711 | -0.14 | 0.03 |
| Trial number^(3)^ | 0.04 | 0.13 | 0.10 | 1 | -0.22 | 0.30 | 0.747 | -0.02 | 0.10 |
| Order^(4)^ | 0.21 | 0.26 | 0.61 | 1 | -0.32 | 0.74 | 0.437 | 0.01 | 0.37 |
| Location of old identity object ^(5)^ | -0.84 | 0.27 | 7.71 | 1 | -1.44 | -0.31 | 0.005 | -1.06 | -0.7 |

(1) Not indicated due to its limited interpretability

(2) Reference category: claw

(3) Z-transformed to a mean of 0 and a standard deviation of 1

(4) Reference category: claw first

(5) During familiarization trials. Reference category: down

Table S6

Experiment 1: results of the intercept-only beta GLMM showing that the proportion of dwell time to the old identity object did not differ from chance level.

| Term | Estimate | Std. Error | z | P |
| --- | --- | --- | --- | --- |
| Intercept | -0.05 | 0.34 | -0.16 | 0.874 |
| Trial number^(1)^ | -0.01 | 0.17 | -0.06 | 0.949 |

(1) Z-transformed to a mean of 0 and a standard deviation of 1

Table S7

Experiment 1: results of the binomial GLMM showing no effect of agent (hand or claw) on the probability to fixate first the old identity object.

| Term | Estimate | Std. Error | χ^2^ | Df | CI min | CI max | P | Min | Max |
| --- | --- | --- | --- | --- | --- | --- | --- | --- | --- |
| Intercept | -0.16 | 0.85 | ^(1)^ | ^(1)^ | -2.81 | 1.77 | ^(1)^ | -0.76 | 0.39 |
| Agent^(2)^ | -0.07 | 0.61 | 0.01 | 1 | -1.68 | 1.65 | 0.909 | -0.38 | 0.5 |
| Trial number^(3)^ | 0.36 | 0.32 | 1.36 | 1 | -0.28 | 1.38 | 0.244 | 0.25 | 0.75 |
| Order^(4)^ | 0.4 | 1.10 | 0.12 | 1 | -1.62 | 3.31 | 0.730 | -0.68 | 1.96 |
| Location of old identity object ^(5)^ | -2.81 | 1.00 | 8.55 | 1 | -8.93 | -1.14 | 0.003 | -4.73 | -2.43 |

(1) Not indicated due to its limited interpretability

(2) Reference category: claw

(3) Z-transformed to a mean of 0 and a standard deviation of 1

(4) Reference category: claw first

(5) During familiarization trials. Reference category: down

Table S8

Experiment 1: results of the intercept-only binomial GLMM showing that the probability that dogs made their first fixation into the old identity object AoI did not differ from chance level.

| Term | Estimate | Std. Error | z | P |
| --- | --- | --- | --- | --- |
| Intercept | -0.23 | 1.35 | -0.17 | 0.863 |
| Trial number^(1)^ | 0.66 | 0.51 | 1.3 | 0.194 |

Table S9

| Term | Estimate | Std. Error | t | Df | CI min | CI max | P | Min | Max |
| --- | --- | --- | --- | --- | --- | --- | --- | --- | --- |
| Intercept | -274.73 | 711.08 | -0.37 | 23.32 | -1564.47 | 1191.3 | 0.716 | -566.33 | 319.46 |
| Agent ^(1)^ | -1083.05 | 505.65 | -2.07 | 24.32 | -2111.9 | -68.58 | 0.049 | -1374 | -840.75 |
| Agent shown first^(1)^ | 121.56 | 905.34 | 0.13 | 16.33 | -1552.25 | 1779.57 | 0.9 | -425.29 | 445.05 |
| Session number^(2)^ | -158.86 | 278.95 | -0.55 | 14.03 | -680.58 | 392.45 | 0.591 | -270.11 | -16.53 |

Experiment 2: results of the LMM investigating the effect of the agent (human or inanimate) on the latency to look at her target during the first familiarization trial of each session.

(1) Reference category: human

(2) Z-transformed

Table S10

| Term | Estimate | Std. Error | t | Df | CI min | CI max | P | Min | Max |
| --- | --- | --- | --- | --- | --- | --- | --- | --- | --- |
| Intercept | -3146.74 | 170.3 | -2.42 | 16.37 | -5496.87 | -650.76 | 0.027 | -3635.94 | -2668.09 |
| Agent ^(1)^ | 1929.43 | 1308.97 | 1.32 | 22.25 | -1053.47 | 4758.57 | 0.199 | 1165.64 | 2716.02 |
| Agent shown first^(1)^ | 2125.23 | 1446.58 | 1.36 | 15.24 | -659.08 | 5158.5 | 0.193 | 1056.7 | 4050.37 |
| Session number^(2)^ | -66.36 | 606.97 | -0.13 | 17.95 | -1302.7 | 1134.04 | 0.899 | -679.5 | 286.58 |

Experiment 2: results of the LMM investigating the effect of the agent (human or inanimate) on the latency to look at the distractor object during the first familiarization trial of each session.

*Note*:

(1) Reference category: human

(2) Z-transformed

Table S11

Experiment 2: results of the binomial GLMM investigating the effect of agent (human or inanimate) on the dogs’ probability to look first at the old identity/new side object during the first four second of the test trials.

| Term | Estimate | Std. Error | χ^2^ | Df | CI min | CI max | P | Min | Max |
| --- | --- | --- | --- | --- | --- | --- | --- | --- | --- |
| Intercept | -3.37 | 1.28 | ^(1)^ | ^(1)^ | -20.57 | -1.83 | ^(1)^ | -5.68 | -2.68 |
| Agent^(2)^ | 1.53 | 1.27 | 1.44 | 1 | -0.9 | 15.38 | 0.231 | 0.9 | 2.32 |
| Session number^(3)^ | 0.16 | 0.41 | 0.16 | 1 | -4.59 | 6.02 | 0.688 | 0 | 0.59 |
| Order of presentation | -0.88 | 1.06 | 0.79 | 1 | -13.22 | 1.23 | 0.373 | -1.48 | -0.47 |

*Note*:

(1) Not indicated due to its limited interpretability

(2) Reference category: human.

(3) Z-transformed

Table S12

Experiment 2: results of the LMM investigating the effect of the agent, the content of the AoI and their interaction on the latency to make a first fixation into one of the AoIs.

| Term | Estimate | Std. Error | t | Df | CI min | CI max | P | Min | Max |
| --- | --- | --- | --- | --- | --- | --- | --- | --- | --- |
| Intercept | 1374.08 | 363.41 | 3.54 | 23.2 | 671.15 | 2114.92 | ^(1)^ | 915.77 | 1641.84 |
| AoI^2^ | 64.06 | 513.04 | 0.12 | 19.01 | -956.39 | 1074.39 | 0.908 | -727.38 | 759.03 |
| Agent^3^ | -1106.94 | 473.87 | -2.17 | 22.47 | -2016.18 | -132.85 | 0.041 | -1638.5 | 70.33 |
| AoI*Agent | 1595.57 | 751.62 | 1.98 | 24.15 | 151.88 | 3086.75 | 0.059 | -48.15 | 2700.58 |

*Note*:

(1) not reported due to its very limited interpretability

(2) reference category: old side/ new identity

(3) reference category: human

Table S13

Experiment 2: results of the beta GLMM investigating the effect of agent on the proportion of dwell time to the old identity object.

| Term | Estimate | Std. Error | χ^2^ | Df | CI min | CI max | P | Min | Max |
| --- | --- | --- | --- | --- | --- | --- | --- | --- | --- |
| Intercept | -0.81 | 0.59 | ^(1)^ | ^(1)^ | -1.98 | 0.39 | ^(1)^ | -5.73 | -0.6 |
| Agent^(2)^ | 0.74 | 0.55 | 1.78 | 1 | -0.33 | 1.8 | 0.182 | 0.5 | 5.63 |
| Session number^(3)^ | 0.23 | 0.23 | 1.26 | 1 | -0.2 | 0.62 | 0.261 | 0.11 | 4.4 |
| Order^(4)^ | -0.51 | 0.39 | 1.69 | 1 | -1.33 | 0.26 | 0.194 | -5.56 | -0.22 |
| Old identity object identity^(5)^ | 0.66 | 0.58 | 1.44 | 1 | -0.53 | 1.73 | 0.23 | 0.48 | 3.35 |

(1) Not indicated due to its limited interpretability

(2) Reference category: human

(3) Z-transformed

(4) Reference category: human first

(5) Reference category: ball

Table S14

Experiment 2: results of the LMM investigating the effect of condition and agent on the dwell time to the agents and their target object after the agents have started to move.

| Term | Estimate | Std. Error | t | Df | CI min | CI max | P | Min | Max |
| --- | --- | --- | --- | --- | --- | --- | --- | --- | --- |
| Intercept | 9740.3 | 1189.9 | 7.77 | 30.61 | 7459.96 | 11985.3 | ^(1)^ | 9153.42 | 10470.89 |
| Condition^(2)^ | -523.24 | 920.46 | -0.56 | 23.79 | -2351.83 | 1271.19 | 0.583 | -862.81 | -117.06 |
| Agent^(3)^ | -2999.82 | 955.09 | -2.97 | 17.32 | -4986.3 | -986.19 | 0.008 | -3469.34 | -2467.87 |
| Session number^(4)^ | -77.32 | 477.87 | -0.16 | 22.28 | -1073.62 | 832.45 | 0.873 | -320.76 | 170.58 |
| Order^(5)^ | 488.28 | 1406.7 | 0.33 | 17 | -2487.48 | 3273.45 | 0.747 | -115.43 | 1137.22 |

*Note*:

(1) Not reported due to its very limited interpretability

(2) Reference category: old side/new identity

(3) Reference category: human

(4) z-transformed to a mean of 0 and standard deviation of 1

(5) Reference category: human sessions first

Table S15

Experiment 2: results of the GAMM investigating the effect of condition and agent on the dogs’ pupil size.

| Parametric coefficients |  |  |  |  |
| --- | --- | --- | --- | --- |
|  | Estimate | Std. Error | t | P |
| Intercept | -366.10 | 127.41 | ^(1)^ | ^(1)^ |
| Condition ^(1)^ | 342.58 | 145.30 | 2.36 | 0.019 |
| Agent ^(2)^ | 68.52 | 145.43 | 0.47 | 0.638 |
| Smooth terms |  |  |  |  |
|  | edf | Ref.df | F | P |
| s(time) | 1.02 | 1.03 | 1.40 | 0.240 |
| s(time)*Condition – new identity | 3.68 | 4.81 | 1.91 | 0.085 |
| s(time)*Condition – new side | 6.83 | 8.46 | 1.23 | 0.363 |
| s(time)*Agent – human | 7.78 | 9.56 | 1.52 | 0.179 |
| s(time)*Agent – inanimate | 0.08 | 0.11 | 0.01 | 0.975 |
| s(Xgaze, Ygaze) | 25.67 | 28.07 | 29.37 | < 0.001 |
| s(time, Event) | 634.31 | 672.00 | 195.27 | < 0.001 |

(1) Not reported due to its very limited interpretability

(2) Reference category: old side/new identity

(3) Reference category: human

Table S16. Power analysis for the binomial model of Exp.2, investigating the effect of agent on the probability to make a first fixation to the old identity object. Power indicates the proportion of converged models whereby a likelihood ratio test of the fixed effect of condition revealed a significant result.

| Performance inanimate agent | Performance human agent | Random slope of condition | Power |
| --- | --- | --- | --- |
| 0.15 | 0.51 | 1.5 | 76% |
| 0.15 | 0.51 | 3 | 47% |
| 0.29 | 0.65 | 1.5 | 69% |
| 0.29 | 0.65 | 3 | 43% |


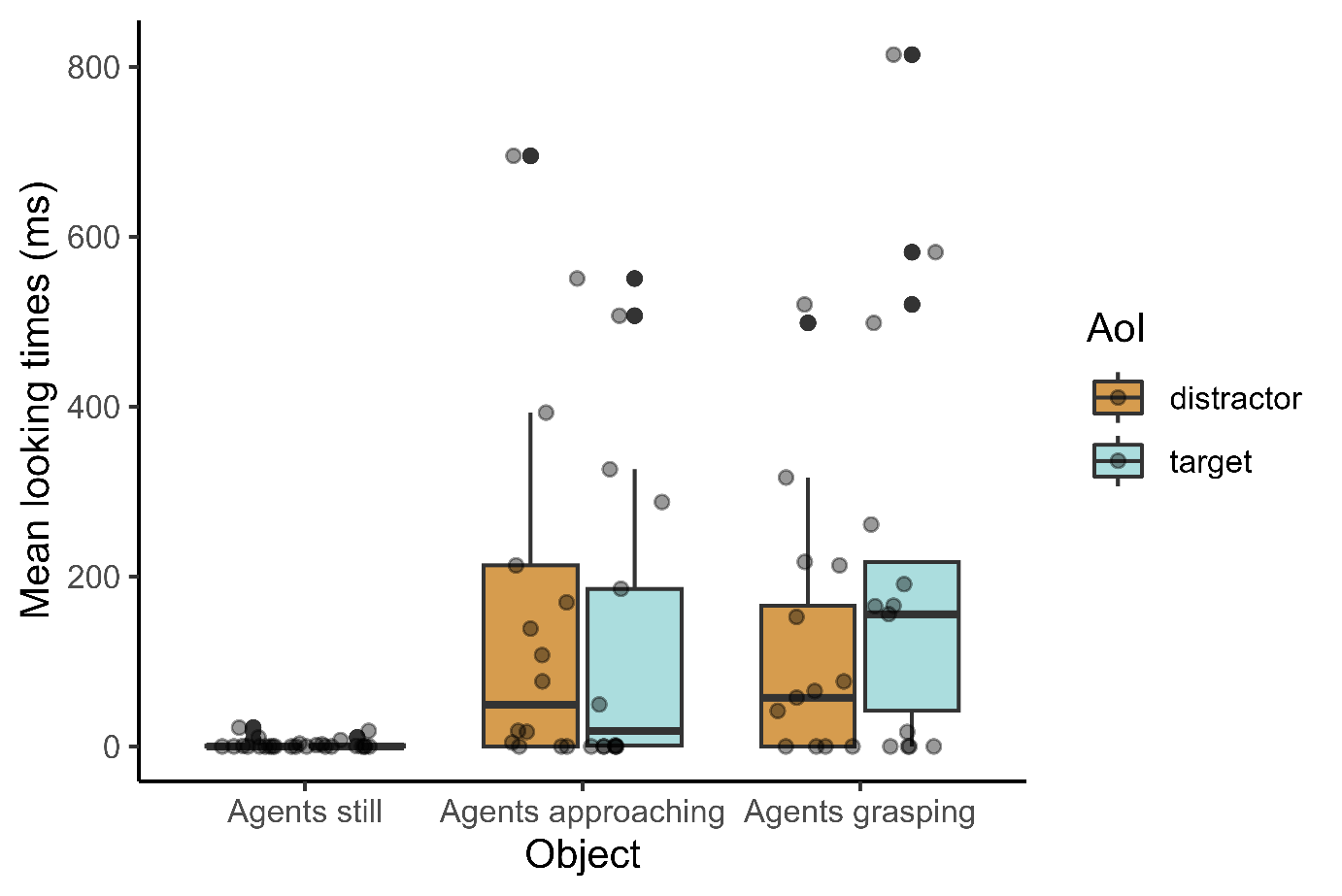
Figures

*Figure S1*. Experiment 1: mean looking times to the objects during familiarization. Looking times are averaged across the first familiarization trial of each of the eight testing blocks (4 trials with the hand and 4 trials with the claw agent). Each dot represents the mean looking time to the target or distractor for one dog in the corresponding period of the video. Looking times to the objects are plotted separately for different interest periods: on the left, when the agents have not started their movement yet (ca. 300 ms); in the middle, when the agents are approaching the target (ca. 1230 ms); on the right, when the agents are grasping the target (ca. 860 ms).

*
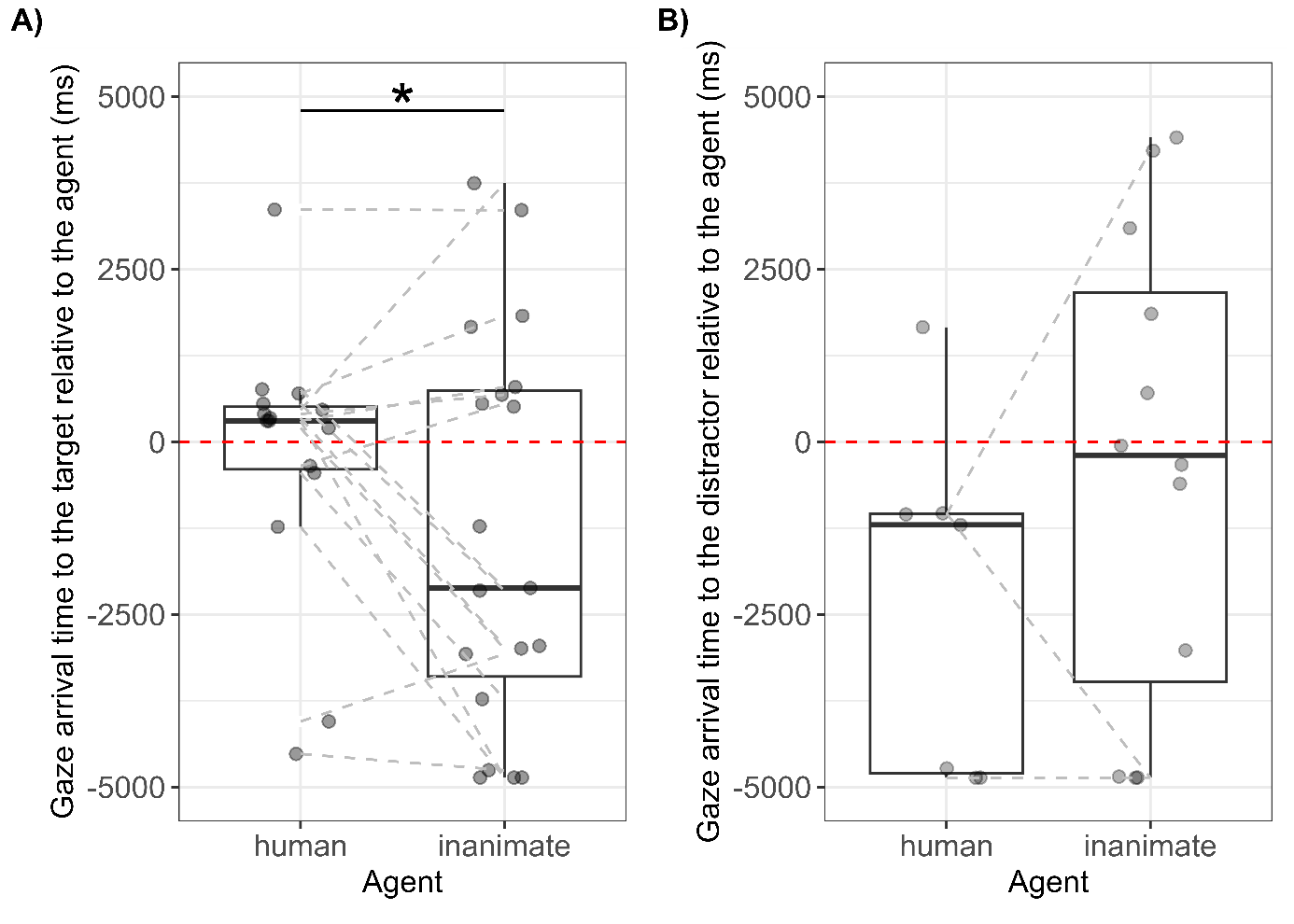
*

*Figure S2*. Experiment 2: gaze arrival times to the target (i.e., the object the agent acted upon; panel A) and distractor (i.e., the other object; panel B) AoIs during the first trial of each familiarization session. The red dashed line indicates the moment when the agent made contact with the objects in the video. Positive values indicate reactive gaze shifts and negative values predictive gaze shifts to the objects. Points indicate the average gaze arrival time for each dog across the two trials with the same agent. The asterisk indicates *p* < .05 (LMMs in Tables S9 and S10).


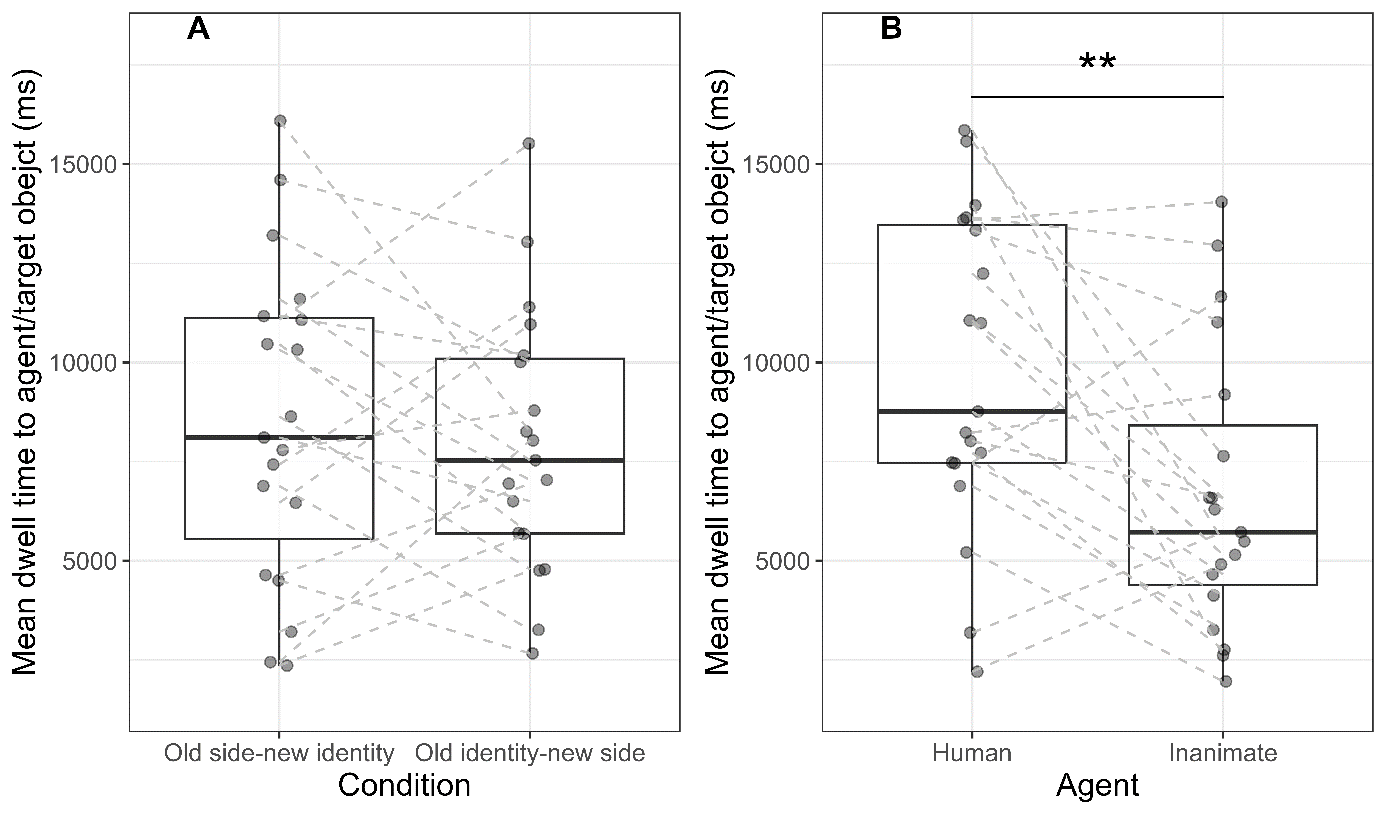


*Figure S3*. Experiment 2: mean dwell time to the agent and the target object (i.e., the object upon which the agent acts). Points show each individual’s average dwell time between trials and lines connect the same individuals between conditions/agents. Dogs looked equally long to the agent/target object between conditions (panel A) but they looked longer to the agent/object when the agent was human than inanimate (panel B). The asterisks in panel B indicate *p* < .01 (LMM in Table S14).

*
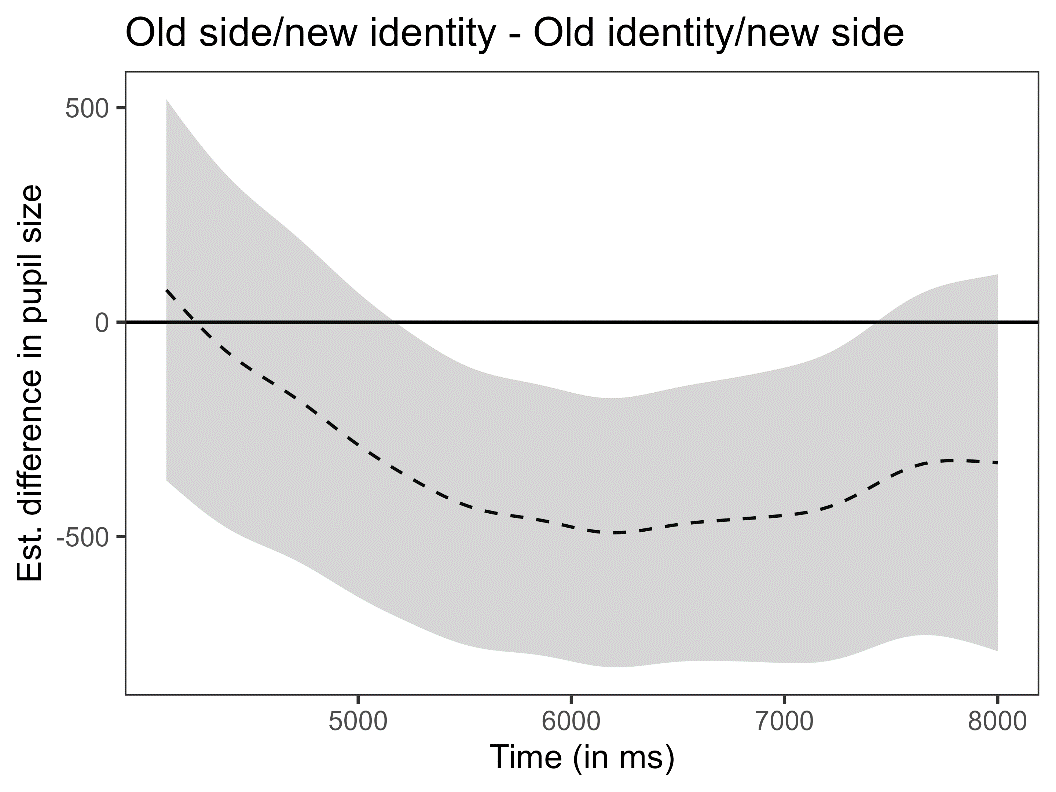
*

*Figure S4*. Experiment 2: difference curve showing the estimated difference in pupil size (dashed black line) between conditions (Old side/new identity – Old identity/new side) based on GAMM predictions. The grey area represents the 95% confidence intervals.

References

Baayen, R. H. (2008). *Analyzing Linguistic Data: A Practical Introduction to Statistics Using R*. Cambridge University Press. https://doi.org/10.1017/CBO9780511801686

Bates, D., Mächler, M., Bolker, B., & Walker, S. (2015). Fitting Linear Mixed-Effects Models Using lme4. *Journal of Statistical Software*, *67*(1). https://doi.org/10.18637/jss.v067.i01

Cannon, E. N., & Woodward, A. L. (2012). Infants generate goal-based action predictions. *Developmental Science*, *15*(2), 292–298. https://doi.org/10.1111/j.1467-7687.2011.01127.x

Field, A. P. (2005). *Discovering statistics using SPSS: (And sex and drugs and rock ‘n’ roll)*. SAGE Publications. http://public.eblib.com/choice/publicfullrecord.aspx?p=743649

Fox, J., & Weisberg, S. (2011). *An R companion to applied regression*. Sage publications.

Harrison, X. A., Donaldson, L., Correa-Cano, M. E., Evans, J., Fisher, D. N., Goodwin, C., Robinson, B., Hodgson, D. J., & Inger, R. (2018). Best practice in mixed effects modelling and multi-model inference in ecology. *PeerJ Preprints*.

Krogh-Jespersen, S., & Woodward, A. L. (2018). Reaching the goal: Active experience facilitates 8-month-old infants’ prospective analysis of goal-based actions. *Journal of Experimental Child Psychology*, *171*, 31–45. https://doi.org/10.1016/j.jecp.2018.01.014

Kuznetsova, A., Brockhoff, P. B., & Christensen, R. H. (2017). lmerTest package: Tests in linear mixed effects models. *Journal of Statistical Software*, *82*(1), 1–26.

Luke, S. G. (2017). Evaluating significance in linear mixed-effects models in R. *Behavior Research Methods*, *49*(4), 1494–1502.

Nieuwenhuis, R. (2012). *Inﬂuence.ME: Tools for Detecting Inﬂuential Data in Mixed Effects Models*. *4*, 11.

Quinn, G. P., & Keough, M. J. (2002). *Experimental design and data analysis for biologists*. Cambridge university press.

R Core Team. (2022). *R: A language and environment for statistical computing.* [Computer software].

Smithson, M., & Verkuilen, J. (2006). A better lemon squeezer? Maximum-likelihood regression with beta-distributed dependent variables. *Psychological Methods*, *11*(1), 54.
